# Likweli: A remarkable new species of *Colobus* monkey from the Lomami National Park, Democratic Republic of Congo

**DOI:** 10.1371/journal.pone.0349857

**Published:** 2026-07-15

**Authors:** John A. Hart, Junior D. Amboko, Julia L. Arenson, Emma R. Horton, Kathryn F. Coates, Jean-Pierre I. Kapale, Mardoché B. Koko, Terese B. Hart, Christopher C. Gilbert, Eric J. Sargis, Kate M. Detwiler

**Affiliations:** 1 Lukuru Wildlife Research Foundation, Kinshasa, Gombe, Democratic Republic of Congo; 2 Division of Vertebrate Zoology, Yale Peabody Museum, New Haven, Connecticut, United States of America; 3 Department of Biological Sciences, Florida Atlantic University, Boca Raton, Florida, United States of America; 4 Lomami National Park, Frankfurt Zoological Society, Frankfurt, Kinshasa, Democratic Republic of Congo; 5 Department of Anthropology, Yale University, New Haven, Connecticut, United States of America; 6 Yale Institute for Biospheric Studies, New Haven, Connecticut, United States of America; 7 Department of Anthropology, Hunter College of the City University of New York, New York, New York, United States of America; 8 PhD Programs in Anthropology and Biology, Graduate Center of the City University of New York, New York, New York, United States of America; 9 New York Consortium in Evolutionary Primatology, New York, New York, United States of America; 10 Division of Paleontology, American Museum of Natural History, New York, New York, United States of America; 11 School of Environmental, Coastal, and Ocean Sustainability, Florida Atlantic University, Boca Raton, Florida, United States of America; Liverpool John Moores University, UNITED KINGDOM OF GREAT BRITAIN AND NORTHERN IRELAND

## Abstract

We describe and name a new species of African monkey, *Colobus congoensis* sp. nov. (Primates, Cercopithecidae), from the interfluve region of the Lomami and Congo (Lualaba) Rivers in east-central Democratic Republic of Congo (DRC). *Colobus congoensis* is a rare and cryptic monkey, poorly known even by local communities bordering its range, some of whom use the vernacular name Likweli for the species. Between 2018 and 2022, 114 field observations were made over an estimated range of 1,700 km^2^. *Colobus congoensis* is largely restricted to high, closed canopy forest on deep clay pediments and islands of terra firme forest, where it co-occurs with two other colobine species (*Piliocolobus parmentieri* and *Colobus angolensis*). *Colobus congoensis* was most frequently observed in small groups (mean = 6.2 individuals), often in mixed-species associations. Mitochondrial and morphological data confirm the attribution of *C. congoensis* to the genus *Colobus* and reveal that it is the sister to *Colobus satanas*, from which it is geographically separated by more than 1,200 km. Comparative analysis of *C. congoensis* vocalizations also reveals structural similarities with *C. satanas* to the exclusion of other *Colobus* species. Among other features, *C. congoensis* is distinguished from *C. satanas* and other *Colobus* species by its small size, a striking orange cream patch surrounding the mouth, philtrum, and portions of the inferior nasal alae on an otherwise black face, and a white perianal patch that is covered with fine white hairs in males and is glabrous in females. We propose a preliminary IUCN Red List classification of Endangered (EN) for *C. congoensis* based on its small range area and population size, coupled with the projected impact of increased hunting pressure and habitat conversion. Protection of Lomami National Park, within which most of the *C. congoensis* range occurs, and engagement of local communities in not hunting the species are the most important actions needed to ensure the conservation of *C. congoensis*.

## 1 Introduction

**Fig 1:**
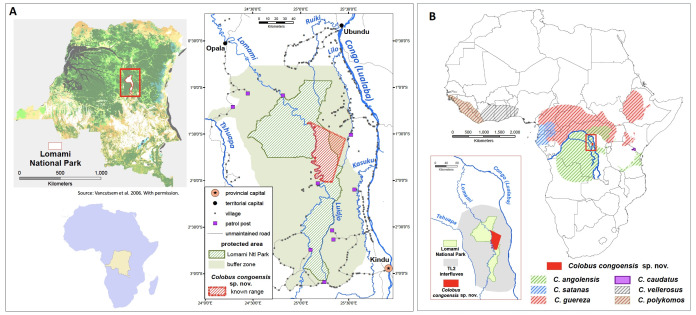
Known range of *Colobus congoensis* sp. nov. **(A)** Distribution in the Lomami National Park and buffer zone, Democratic Republic of Congo. Land cover map reprinted from Vancutsem *1:3,000,000* – Explanatory note*.* ISBN 2-87463-016-0 under a CC BY license, with permission from Presses Universitaires de Louvain, original copyright 2006. Additional sources NASA landsat (2010) open source. **(B)** Distribution of previously described species of the genus *Colobus* based on data from IUCN Red List [2–7]. The red rectangle locates the insert map of the known range of *Colobus congoensis* sp. nov. in relation to the Lomami National Park and the Tshuapa-Lomami-Lualaba (TL2) interfluves.

We report the discovery of a new species of African primate from the eastern basin of the Lomami River and adjacent basin of the Upper Congo (Lualaba) River, Democratic Republic of Congo (DRC). The new species is here assigned to the subfamily Colobinae, genus *Colobus* within the Cercopithecidae. The genus *Colobus* is endemic to tropical Africa, with six currently recognized species [1]. This new species is genetically distinct and characterized by a suite of unique morphological features as well. It is restricted to an apparently relict range in the Lomami-Lualaba interfluve, bordered on the west by the Lomami River and on the east by the Lilo River and a mosaic of seasonally flooded and terra firme forest (Fig 1).

Reports of new extant catarrhine primate species are rare. A number of taxa have been elevated from subspecies to species in the last 75 years, including at least four hominoids and six cercopithecids [8–17, see also 18,19]. Since 1950, eight catarrhine species represented by populations previously unknown to science have been reported, including the Lesula, *Cercopithecus lomamiensis,* which, like the new *Colobus* described here, is restricted to the Tshuapa-Lomami-Lualaba (TL2) interfluve region [20–28]. The new *Colobus* species represents only the fifth new species of African monkey to be discovered in the past 75 years.

This paper presents a diagnosis and description of the new species, with Likweli recommended as its common name. Among living African colobines, Likweli most closely resembles *Colobus satanas*; the latter is restricted to west-central Africa and Bioko Island in the Gulf of Guinea and is separated from the new species by over 1,200 km (Fig 1). We present morphological, genetic, and acoustic evidence to establish the distinctiveness of the new *Colobus* species, and we present preliminary data on its distribution, abundance, ecology, and behavior. We discuss the hydrological and landscape history of the Congo’s central basin and the Lomami-Lualaba interfluve as relevant to the likely range limitation and biogeography of this new species. Finally, we provide a preliminary assessment of the conservation status for the new species. This discovery confirms the importance of the Lomami National Park and its buffer zone, located in the TL2 interfluve region, for the conservation of the new colobine along with a diverse primate fauna endemic to the DRC.

## 2 Materials and methods

### 2.1 Ethics statement

Field observations, community engagement, and the acquisition and export of specimens were conducted under the authorization of the Congolese Institute for Nature Conservation (ICCN), the DRC government agency responsible for protected areas and with jurisdiction over their wildlife. ICCN operations are guided by the Congolese Law 08/08 of 2008 concerning Forest Classification, the National Strategy for the Conservation of Biodiversity in the Protected Areas of DRC (2012), and the Law 14/003 relative to Nature Conservation of 2014. These require ICCN to assure adequate outreach and dialogue with communities whose lands border protected areas. Information provided by residents of these settlements concerning the new *Colobus* monkey, *C. congoensis* (Likweli), was collected fortuitously or secondarily during village gatherings to raise awareness about the park. The gatherings were authorized by the ICCN, but organized with local administrative and traditional authorities. Typically, during a village visit by representatives of the ICCN, a chief or elder would call a village meeting where park rules as well as rights and responsibilities of the community members relative to the park and buffer zone were discussed. Villager attendance was not required, people were free to come and go, and could volunteer information or not. In these sessions, ICCN was particularly concerned with the communities’ perception of the buffer zone in which they lived, the composition of its fauna and how communities exploited it or were impacted by it. In this context, ICCN would ask questions about rare species including the presumed new *Colobus* species. For instance, the information regarding the rare occurrence of Likweli in the eastern portion of its range was acquired during community sessions explaining the park limits and the extent of the buffer zone before mobilizing community members for the participatory demarcation process in the Mituku sector of the buffer zone.Field work associated with the discovery of this species was conducted under partnership contracts between ICCN and the Lukuru Foundation (2011 – 2019) and between ICCN and the Frankfurt Zoological Society (FZS) from 2019 to present. The Lukuru Foundation led explorations of the Tshuapa-Lomami-Lualaba interfluve region (TL2) and established Lomami National Park in collaboration with ICCN, while FZS supports the ongoing co-management and protection of the park and buffer zone in partnership with ICCN.

Specimens of deceased individuals of the new colobine species were confiscated from hunters during ICCN patrols in the national park in April 2021. We acquired samples from *C. angolensis* killed in the buffer zone and destined for illegal bushmeat trade. Confiscations followed the ICCN Code of Conduct for park guards. Acquisition of the specimens for scientific description was authorized by the ICCN. Specimens were exported under CITES authorization and imported into the United States of America under permits by the Centers for Disease Control and Prevention and US Fish and Wildlife Services. Non-exported specimens are housed at the University of Kisangani’s Centre de Surveillance de la Biodiversité (CSB) in accordance with CSB procedures. Non-invasive field observations were made in Lomami National Park by field teams composed of project scientific staff, ICCN eco-guards, and local community monitors. Additional information on the species, including its distribution and hunting threats, was provided by residents in communities living within the buffer zone who were interviewed in the context of community conservation projects.

### 2.2 Local knowledge

We evaluated local knowledge on the occurrence and ecology of the new species by conducting interviews with residents and hunters familiar with the area comprising its range and vicinity. Interviews were conducted in 52 villages in the Sectors of the Mituku Basikate, Mituku Bamoya, and Walengola-Babila in Tshopo Province and in the Balanga Sector in Maniema Province. We asked interviewed individuals to describe primates that occurred in their area of activity and to provide vernacular names. For likely observations of the new *Colobus* species*,* we asked informants to provide detailed descriptions of the monkey, information on behavior and ecology, locations where they encountered them, and if they were a target for hunting. We accepted observations of the new species if the reports included descriptions of diagnostic pelage and facial features. When specific locations for sightings were identified, and when logistics permitted, we invited informants to accompany field teams to return to the site to search for the animals.

### 2.3. Inclusivity in global research

Additional information regarding the ethical, cultural, and scientific considerations specific to inclusivity in global research is included in the Supporting Information (S1 Text).

### 2.4. Morphology

#### 2.4.1 Specimens examined.

Three specimens (two adult females and one adult male) were sampled for genetic analysis and provided the basis for morphological description of the new species. In addition, we sampled nearby populations of *C. angolensis angolensis* for comparative genetic analysis (tissue samples of one male and one female). Specimens of the new species were skinned and skeletonized in the field for transport to the University of Kisangani’s Centre de Surveillance de Biodiversité (CSB) Zoological Museum. At CSB, the skins were treated with alum and air dried. The skeletons were cleaned with a solution of carbon tetrachloride, deionized salt, and deionized water, and then treated with hydrogen peroxide. Tissue samples collected in the field were stored in RNAlater (Applied Biosystems, Foster City, California) and 94% ethanol and kept in cool, dark locations until shipped. Skin, skeletal, and tissue collections were shipped and exported to the Yale Peabody Museum (YPM), New Haven, Connecticut, with additional tissue samples stored at CSB and on loan from YPM to Florida Atlantic University (FAU).

#### 2.4.2 External morphology.

Field examinations included standard external measurements [29]: total length, tail length, length of hind foot, length of ear pinnae, and body mass. Body mass was measured with a spring balance accurate to 0.1 kg. Photographic documentation was made of external morphology for all individuals before preparation of specimens. Photos of living animals observed in the field provided additional documentation of the species’ physical appearance. We compared the new colobine’s morphology with that of other African colobine (subtribe Colobina) species using museum specimens and photos available on iNaturalist, IUCN Red List, and other verified sources on the internet.

#### 2.4.3 Craniodental and skeletal measures and analysis.

Skeletal specimens of 13 extant African colobine species were sampled for comparative cranial and dental analysis (n = 182; Table 1), following the taxonomy used by the IUCN [1]. Five species of *Colobus* were sampled (with multiple subspecies of *C. guereza* and *C. angolensis*), excluding the recently elevated and skeletally rare Mt. Kilimanjaro Guereza, *Colobus caudatus* (a close relative and former subspecies of *C. guereza* [15]). Seven species of *Piliocolobus* were also sampled, as well as the monotypic *Procolobus verus*. Wild specimens were included whenever possible, but a few non-pathological captive specimens were included to increase the sample size for *Piliocolobus kirkii*. Only adult specimens were measured for craniomandibular metrics, as defined by full M^3^/_3_ eruption. Dental metrics on erupted adult teeth in juvenile specimens were included to increase sample sizes of *C. vellerosus* and *C. satanas*. A complete list of sampled specimens, including subspecific identifications and calculated indices for each specimen, is provided in Table A in S1 File.

**Table 1:** Comparative skeletal sample used in morphometric comparisons.

Species	F	M	?	Total
*Colobus satanas*	9	9	0	**18**
*Colobus angolensis*	11	13	0	**24**
*Colobus guereza*	14	13	0	**27**
*Colobus polykomos*	10	12	0	**22**
*Colobus vellerosus*	2	1	2	**5**
*Piliocolobus badius*	10	11	0	**21**
*Piliocolobus foai*	0	1	0	**1**
*Piliocolobus gordonorum*	2	0	0	**2**
*Piliocolobus kirkii*	8	1	0	**9**
*Piliocolobus oustaleti*	9	10	0	**19**
*Piliocolobus rufomitratus*	1	0	0	**1**
*Piliocolobus semlikiensis*	6	3	0	**9**
*Procolobus verus*	12	12	0	**24**

F = females; M = males; ? = unknown sex.

Nine skeletal indices were calculated to compare the new colobine with extant African colobines, capturing aspects of size and shape in the cranium, mandible, and dentition (Table 2). To control for possible allometric scaling effects, Mosimann shape indices of the craniodental features were tested against the geometric mean of 17 linear cranial measurements as a proxy for specimen size [30–32]. Indices were considered allometrically influenced if they had a significant and strong correlation with size (cranial geometric mean) indicated by a Pearson’s correlation coefficient |r| > 0.5 [32–34]. Absence of a strong and significant correlation between size and shape suggests isometry of the shape variable within the sample. Indices that scale allometrically are figured as bivariate scatter plots to compare specimens at a similar size. Non-allometric (isometric) indices are presented as boxplots, and statistically significant differences were assessed among genera and species with ANOVAs and Tukey’s Honestly Significant Difference (HSD) post-hoc pairwise comparisons.

**Table 2:** Description of craniomandibular measurements and indices used in morphometric comparisons.

Index	Definition/description
Cranial size (GMean)	Geometric mean of 17 linear measurements: basion–bregma, basion–nasion, glabella–inion, nasion–prosthion, nasion–rhinion, prosthion–inion, interorbital breadth, orbit height, orbit width, minimum depth of zygomatic, external breadth of palate at M^1^, prosthion–staphylion, maxillary molar row length, basion–prosthion, biporionic breadth, maximum length of occipital condyle, & mediolateral width of foramen magnum
Airorhynchy	Angle of cranial flexion at glabella between inion and prosthion, calculated between glabella–inion chord, glabella–prosthion chord, and prosthion–inion chord
Petrous shape	Anteroposterior length of petrous from jugular foramen to petrous apex divided by mediolateral width of petrous over the carotid canal
Palate shape	Prosthion–staphylion chord divided by external palate breadth at M^1^
Relative corpus depth	Depth of corpus at level of M_1_/M_2_ divided by GMean
Relative I^1^ breadth	Mediolateral breadth of I^1^ crown divided by M^1^ MD length
Relative canine area	Maxillary canine area (BL × MD) divided by M^1^ area (mesial BL × MD)
Relative P^4^ size	P^4^ MD length divided by M^1^ MD length
M_2_ shape	M_2_ MD length divided by mesial BL breadth

Abbreviations: MD = mesiodistal; BL = buccolingual. See Table A in S1 File for complete comparative dataset of these indices.

Postcranial morphology will be described in a separate, functional analysis. For the current descriptions, we provide craniodental measurements and basic limb indices (i.e., intermembral index) in the supplementary information (Table B in S1 File).

### 2.5 Genetics

DNA was extracted from tissue samples of three individuals of the new *Colobus* species and two *C. angolensis angolensis* individuals using the DNeasy Blood & Tissue Kit (Qiagen 69504; Germantown, MD) following manufacturer's protocols. A 4,090 base pair (bp) region of the mitochondrial genome was amplified, including the NADH3, NADH4L, NADH4, and NADH5 genes [35]. This marker has been used in previous studies to investigate mitochondrial phylogenies of African and Asian colobines [35,36] and allows for comparative analysis between the new species and all recognized *Colobus* species. We used PCR primers from Ting [35] and designed additional sequencing primers (available upon request) based on known African colobine templates. Sanger sequencing was performed by the Molecular Cloning Laboratories (San Francisco, California), and sequence chromatograms were inspected by eye and assembled using Geneious R11 (v11.0.5; https://www.geneious.com). Additionally, we obtained orthologous sequences from Asian and African colobine species via the National Center for Biotechnology Information GenBank database (S1 Table). Novel sequences generated in this study have been archived in GenBank (S1 Table; https://www.ncbi.nlm.nih.gov/nuccore/?term=Likweli). Only unique haplotypes were included in the final alignment.

To infer a phylogeny, we assembled and annotated the mitochondrial regions of all the samples with MitoFinder/1.4.1 [37], using a published *Piliocolobus badius* mitochondrial genome as reference (NC_008219) [38]. Each mtDNA region was then divided into 13 partitions: 1st, 2nd, and 3rd codon positions of four protein-coding genes and a concatenated sequence of four tRNA, which were individually aligned using MAFFT/7.407 [39]. The best evolutionary model was evaluated using jModelTest [40]. A maximum likelihood tree was constructed in RaxML [41], using the GTR + F model of substitutions and performing 1,000 rapid bootstraps.

Divergence dates were estimated using a Bayesian phylogenetic analysis conducted with MCMCTree, as implemented in PAML v4.9j [42]. We used a single sample per species or subspecies for all monophyletic groups and included one representative per clade in cases of paraphyly. We applied a fossil-calibrated prior to the Colobina–Presbytina split based upon de Vries and Beck [43], using a uniform distribution with a hard minimum bound at 8.125 million years ago (Ma) and a soft maximum bound at 15 Ma [see also 44]. To facilitate comparison with previously published divergence estimates for the *Colobus* clade, we also performed analyses using a secondary calibration for the African-Asian colobine split, constraining this node to 9.42–12.28 Ma based on the divergence estimate reported by Perelman et al. [45] and subsequently used as a calibration point in Roos and Zinner [36]. For all MCMCTree analyses, we employed the correlated-rates clock model, discarded the first 10,000 iterations as burn-in, and sampled every 50 iterations until a total of 20,000 post-burn-in samples were obtained.

### 2.6 Vocalizations

We recorded six roaring events from the new colobine species in Lomami National Park during 2020 and 2021 using a Canon digital camera (n = 4), Zoom H5 4-input digital recorder with a Sennheiser MKE 600 shotgun microphone (n = 1), and a cell phone (n = 1). It was not possible to distinguish the caller’s sex. However, roaring is more common in adult males in other *Colobus* species [46]. Recordings were collected at locations spaced 3–15 km apart, likely from individuals in different social groups. We could hear more than one individual calling in some recordings. Three recorded events produced vocalizations that were suitable for bioacoustic analysis. We compared recordings of the new *Colobus* species to *C. satanas* vocalizations from Lope National Park, Gabon and from Bioko Island, Equatorial Guinea. Structural components of the new *Colobus* species and *C. satanas* roars were also compared with published roars of other *Colobus* species [46–49].

We uploaded recordings into Audacity 3.7 software (open source, Muse Group) and applied a noise reduction filter (12 decibels). The filtered recordings were analyzed with Raven Pro software (Cornell Laboratory of Ornithology, Ithaca, New York) using methods adapted from Schel et al. [49], Charif et al. [50], and Fuller [51]. To characterize the new *Colobus* roars and compare them to other *Colobus* roars, we measured basic call features including pulse rate (pulses/second), peak fundamental frequency (Hz), and presence or absence of frequency modulation. For a more detailed comparison between high-quality recordings of the new *Colobus* and *C. satanas* roars specifically, we analyzed 23 temporal and acoustic call features (Table A in S2 File) [51].

We generated spectrograms using a 512-point fast-Fourier transform and a Hann window function to measure the morphology of the vocalizations. We generated a power-time oscillogram to determine temporal limits and a power frequency spectrum using a 1024-point fast-Fourier transform and a Hann window to determine frequency measurements and frequency limits. The noise floor in the power frequency spectra was determined for each file using a region of the audio with no signal and only background noise. We used summary statistics and principal component analysis (PCA) to compare roar phrases between the new *Colobus* and *C. satanas*.

### 2.7 Field surveys

#### 2.7.1 Distribution, relative abundance, and group composition.

Between 2018 and 2022, we used five methods to record encounters with unhabituated groups of the new *Colobus* species in Lomami National Park and the adjacent buffer zone: terrestrial surveillance patrols, line transect inventories, dawn call counts, riverine patrols, and directed searches guided by local informants. All surveys were conducted by field teams led by technicians trained to identify and document observations of primates and who had experience with the new *Colobus* species specifically (Text A in S3 File, Table A in S3 File).

Survey teams initially detected most primates by auditory indicators: vocalization or movement of vegetation. Observers left the survey track line to make visual contact with detected groups and recorded the following data when possible: species identity, number of individuals, age class, position in the vegetation stratum, forest type, food items eaten, and other behaviors observed opportunistically. Locations of all primate encounters were documented by handheld GPS receivers, and photos were taken when possible.

Terrestrial surveillance patrols and riverine patrols were conducted with ICCN park guards to provide comprehensive coverage of the park and adjoining buffer zone, serving both law enforcement and biomonitoring objectives. Between 2020 and 2022, deployment of terrestrial surveillance patrols was increased in the areas where the new *Colobus* species had been documented to increase the possibility of further detections. Search effort for the new species on terrestrial surveillance patrols was evaluated by Geographic Information System (GIS) software within a 5 × 5 km grid that covers the park and buffer zone. Grid cells were defined as having high surveillance patrol coverage if the cell was visited by at least 3 surveillance patrols per year between 2020 and 2022 and accumulated a total patrol track of at least 30 km.

Line transect surveys were conducted between 2020 and 2022 and covered inventory blocks that were independent of the surveillance patrol grid. Transect inventory blocks varied in size from 53 km^2^ to 129 km^2^ and were designed to cover major habitats within the range of the new *Colobus* species, including areas of both high and low rates of detections on surveillance patrols. Survey effort within a line transect inventory block was defined as the summed transect distance on replicate surveys. Relative abundance (encounter rate) of the new *Colobus* and other primate species was determined as the number of encounters within a block divided by the summed transect distance covered during replicate surveys.

Dawn surveys of primate vocalizations (loud calls) were conducted at 351 locations across the new *Colobus* species’ range area between 2020 and 2022. Surveys were conducted for 30-minute periods by 3 concealed stationary observers, starting between 05:30 and 6:15. Each team had at least one person familiar with the new *Colobus* species’ roars who was able to distinguish these from the roars of sympatric *C. angolensis.* For all identifiable primate vocalizations heard, observers recorded compass direction from the listening point, number of calling bouts in the session, and relative audio clarity of the call.

#### 2.7.2 Habitat description and use.

Habitat classification of encounters with the new *Colobus* species was determined by superimposing observation locations on a map of topographic and substrate classes in the park and buffer zone with the addition of the forest type as determined in the field at the location of the observation. Topographic and substrate classification of the Lomami National Park and buffer zone is based on two inputs: 1) a land cover classification of 2010 Landsat imagery, and 2) geo-referenced field data on topography, elevation, seasonal inundation, and predominant soil type (clay or sand). Topographic descriptions follow Guillocheau et al. [52] for Congo Basin Neogene landforms and Bwangoy et al. [53] for seasonally inundated sites. Habitat classes identified in the range area are described in Table 3.

**Table 3:** Habitat classes within the range of the new *Colobus* species.

Habitat class	Predominant Vegetation	Elevation above sea level (m)	Substrates	Relief and topography	Soils	Notes
Low relief forest	Mature, closed canopy forest	350 - 500	Terra firme	Low relief and topography	Varied: clays to sands	Soil type varies locally
Pediment forest	Mature, closed canopy forest	350 - 500	Terra firme	Localized high relief, varied topography	Clays dominant	Frequent incised small stream valleys
*Sende* mosaic	Closed to open canopy forest	350 - 450	Mosaic upland and seasonally waterlogged	Low relief and topography	Extensive areas white sands	Localized variation, upland soils, clays to sands
Riverine forest	Mature, closed to open canopy forest	350 - 450	Seasonally inundated	Low relief and topography	Varied: clays to sands	Adjacent to water courses, seasonal flowing water
Regenerating forest	Recent secondary forest	350 - 500	Terra firme	Low relief and topography	Varied: clays to sands	Areas of tree blowdowns and fallow gardens

### 2.8. Nomenclatural acts

The electronic edition of this article conforms to the requirements of the amended International Code of Zoological Nomenclature, and hence the new names contained herein are available under that Code from the electronic edition of this article. This published work and the nomenclatural acts it contains have been registered in ZooBank, the online registration system for the ICZN. The ZooBank LSIDs (Life Science Identifiers) can be resolved and the associated information viewed through any standard web browser by appending the LSID to the prefix “http://zoobank.org/”. The LSID for this publication is: urn:lsid:zoobank.org:pub:AAB9196D-DC31–4172-A218-CE924AE3BC65. The electronic edition of this work was published in a journal with an ISSN, and has been archived and is available from the following digital repositories: PubMed Central, LOCKSS.

## 3 Results

### 3.1 Discovery and local knowledge

The first evidence of the new species came in 2008 during exploration of the Lomami Basin by members of the Lukuru Foundation, in an area that later became Lomami National Park. Ashley Vosper and Bernard Ikembelo photographed an unidentified monkey in a high forest canopy on the east bank of the Lomami River in the Courbure Sector, named for six large meanders of the Lomami River. Although the photograph revealed only part of the animal, the field team strongly suspected that this was not any of the monkey species they already knew from the region. Following the initial sighting, nothing more was reported until November 2018, when Jean Pierre Kapale led a surveillance patrol in the Courbure Sector and photographed a black monkey with pale markings around its mouth and a white perianal patch. These characters were unlike those of any known primate species in the area. Over the next 10 months, Kapale and his team, using surveillance patrols and directed searches accompanied by local field assistants, found and photographically documented the monkey seven more times in different locations. Comparisons of the photos taken in 2008 and in 2018–2019 showed that the monkey Kapale encountered was the same species as the previously unknown monkey seen a decade earlier. Following Kapale’s reports, we conducted a review of photographs documenting primate sightings in earlier surveillance patrols by other teams. We found that the same monkey had been seen and photographed in August 2018 in an area 35 km north of Kapale’s discoveries, but that the monkey had been misidentified in the patrol report and never announced at that time.

The newly discovered *Colobus* species is not well known by local communities, even within the vicinity of its range. This contrasts with the extensive knowledge of other primates in these communities. Residents in only eight out of the 52 surveyed localities bordering the new species’ range reported knowledge of the primate and could accurately describe it. In one interview, a hunter who had once opportunistically killed the monkey described it but could not give it a local name. Another hunter described the species’ strong odor. Eventually, the people of the Balanga ethnic group living in the buffer zone west of the Lomami River and bordering the species’ range gave Jean Pierre Kapale the vernacular name, Likweli. The Mituku local communities, who occupy the eastern limits of the range, referred to the species as *kasaba nkoni*, which means “the branch shaker.” The species was described as quiet and cryptic with a localized distribution. Several informants stated that its calls, reported mostly to be heard at dawn, resembled the roars of *C. angolensis*.

### 3.2 Diagnosis and description

#### 3.2.1 Systematics.

Family Cercopithecidae Gray, 1821

Subfamily Colobinae Blyth, 1863

Subtribe Colobina Blyth, 1863

Genus *Colobus* Illiger, 1811

***Colobus congoensis*** J. Hart, Amboko, Arenson, Horton, Coates, Kapale, Koko, T. Hart, Gilbert, Sargis, and Detwiler, **sp. nov.** urn:lsid:zoobank.org:act:8F7FB2EC-5E42-4517-9D8C-634A2F7CBD5D

**Holotype**: Yale Peabody Museum, YPM MAM 17307 (Field Catalog JH30), adult male, skin and skeleton.

**Paratypes**: Yale Peabody Museum, YPM MAM 17306 (Field Catalog JH28) and YPM MAM 17308 (Field Catalog JH31), two adult females, skins and skeletons.

**Type Locality**: Lomami National Park, Democratic Republic of Congo, Tshopo Province, Biondo Sector, east bank of the Lomami River, S1.70190°, E25.13970°.

**Etymology**: The nominal *congoensis* refers to the species range limited to the Democratic Republic of Congo. Likweli, the vernacular name given to the species by communities bordering its range, is the recommended common name.

**Fig 2:**
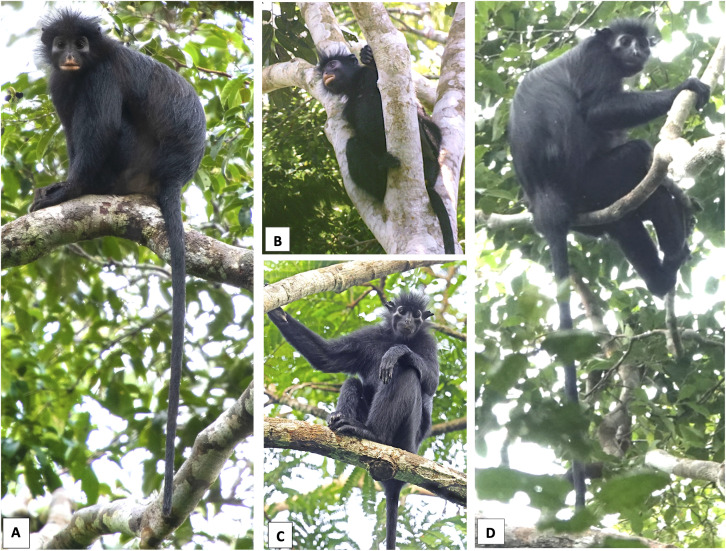
*Colobus congoensis* and *C. satanas* in life. **(A & B)** *C. congoensis*, Lomami National Park, Democratic Republic of Congo. Photo credits: (A) Daniel Rosengren, (B) Bravo Bofenda. **(C & D)** *C. satanas*, Lope National Park, Gabon. Photo credits: (C) Martin Royele/Royele Safaris, (D) Barna Takats. Photos used with permission.

**Fig 3:**
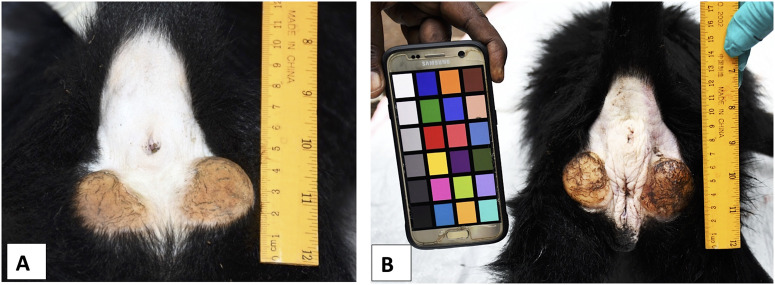
*Colobus congoensis* perianal patch. **(A)** Adult male (YPM MAM 17307). **(B)** Adult female (YPM MAM 17308).

**Diagnosis and Description**: A small, long-tailed *Colobus* monkey that exhibits little sexual dimorphism in overall pelage coloration. The pelage of the limbs and torso is black. Hair length is longest on the upper shoulders and back (Color 89 in [54]), producing a sheen in some lighting and a cape-like pelt in some individuals. Body hairs are relatively short (~9–12.5 cm long in the male; ~ 6–8 cm long in females at mid-dorsum). The tail is largely black. Older individuals may show gray hairs (Glaucous Colors 79–80 in [54]) along the caudal dorsum and the tail, as seen in YPM MAM 17308, which also exhibits heavily worn dentition in the associated cranium, indicating an advanced age for this individual (Text A and Fig B in S4 File). The single male examined had a small, black terminal tail tuft in the fresh specimen, not seen on the two females. The head in both sexes is framed by long black hairs (~4–6 cm) on the forehead and sides of the face. The dark glabrous face is marked in both males and females by a prominent patch of bare skin that is pinkish to orange-cream in color, surrounding the mouth and extending from the upper lip and philtrum to the inferior portions of the nasal alae. The extent of this pale patch is variable, in some individuals covering much of the lower face below the nares and in others restricted to the circumoral region and philtrum. The bare skin over the pronounced zygomatic arches and temporal region is dark gray, contrasting with the black skin on eyelids and around the eyes, and giving the face a mask-like appearance (Fig 2). Ear pinnae are large and black with crenulated and folded margins. Both sexes have a white perianal patch (9 × 5 cm in the male; 12 × 6 cm in the female) between pale orange callosities. The patch is largely glabrous in the females whereas it is sparsely covered with fine white hairs in the male (Fig 3, S2 Table). The scrotum and penile sheath are dark gray, and the penis is pinkish white. Field measures are given in S3 Table.

**Fig 4:**
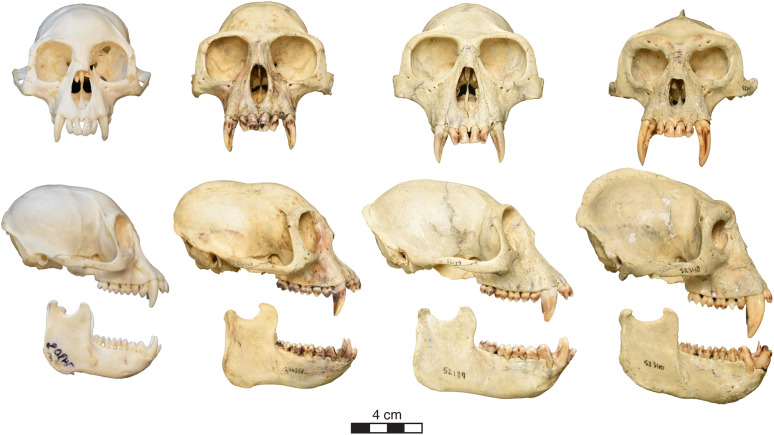
Holotype of *C. congoensis* compared with other African colobines. Left to right: *C. congoensis* holotype male skull (YPM MAM 17307), *Colobus satanas* male skull (AMNH M-236358), *Colobus angolensis* male skull (AMNH M-52189), and *Piliocolobus oustaleti* male skull (AMNH M-52340). Top row: anterior view of cranium; middle row: right lateral view of cranium; bottom row: right lateral view of mandible.

The cranium of *C. congoensis* is airorhynchous with a narrow and elongated basicranium (Fig 4; Figs A-C in S4 File). The interorbital pillar is broad, and in profile forms a continuous curve with a nearly vertically oriented frontal squama. The anterior portion of the neurocranium displays modest frontal “bulging” or “saddle-shape” in lateral view. The neurocranium is globular with widely spaced temporal lines originating from the lateral margin of the supraorbital torus. The male individual YPM MAM 17307 has slightly raised temporal lines and a moderately developed nuchal crest; females (YPM MAM 17306 and YPM MAM 17308) are more gracile (see Text A in S4 File and Figs A-C in S4 File for further descriptions and photographs of individual specimens). The rostrum is highly abbreviated with moderate subnasal prognathism, and the malar is shallow. The orbits are bounded superiorly by a thin torus restricted to the lateral ~3/4 of the orbit. The mandibular corpus is shallow and deepens slightly posteriorly. The gonion is clearly expanded in the male individual but not greatly expanded in the female individuals. The symphysis is sloping, unmarked by mental ridges or a median mental foramen. The corpus lacks lateral buttressing in the form of *prominentia laterales*. The ramus is tall and vertically to slightly posteriorly oriented, with a shallow coronoid notch.

**Fig 5:**
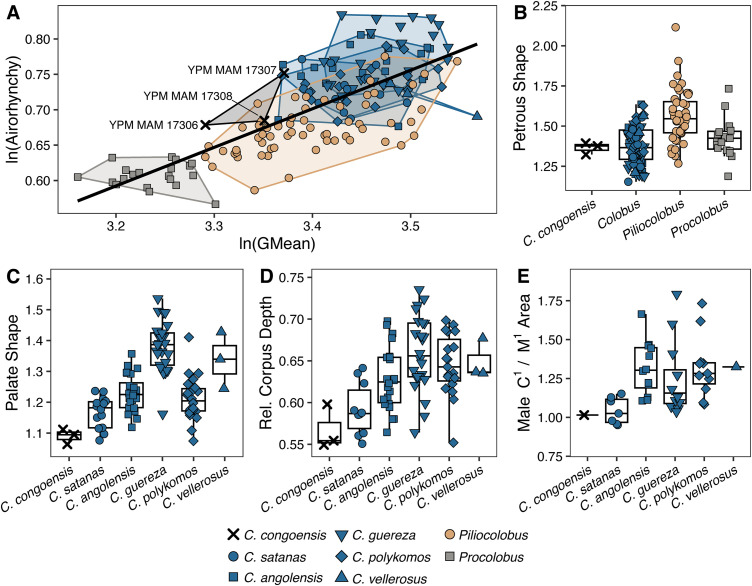
Craniomandibular indices among African colobines and within *Colobus.* **(A)** In cranial flexion (airorhynchy), *C. congoensis* is more similar to *Colobus* than *Piliocolobus* of similar size. **(B)** The petrous pyramid is shortened in *C. congoensis*, more similar to that of *Colobus* or *Procolobus* than to the elongated petrous of *Piliocolobus*. **(C)** In palate shape, *C.* *satanas* and *C. congoensis* share a relatively short and broad palate. **(D)** A relatively shallow mandibular corpus differentiates *C. congoensis* and *C. satanas* from other species of *Colobus*. **(E)** Male relative canine size is small in *C. satanas* and the single male specimen of *C. congoensis*. Skull indices are described in Table 2.

Dentally, the molars are typically colobine, characterized by tall cusps, low crowns, and minimal basal flare with distinct mesiobuccal clefts in the lower molars. No accessory cuspules are observed on the molars. The upper P^3^ lacks a protocone, as is typical of African colobine monkeys, but the P^4^ is relatively small compared to that of other species of *Colobus*. Although only one male specimen is known, male upper canine size is relatively small, similar to that of *C. satanas* males (Fig 4; Fig 5E). The female canines are also relatively small and not masculinized, unlike the female canines of *C. guereza*. Overall canine dimorphism, however, is within the range of other *Colobus* species (Table A in S1 File). The lower P_4_ metaconid is subequal to or slightly taller than the protoconid. The upper incisors are small and typically colobine, unlike the enlarged incisors of *C. satanas*. The lower incisors have enamel on the lingual aspect and a distinct prong on the distal margin of the lateral incisors.

#### 3.2.2 Comparisons among the Colobina.

Among colobinans, *C. congoensis* shares black pelage and a number of craniodental similarities with *Colobus* to the exclusion of *Piliocolobus* and *Procolobus* (Fig 5; Fig D in S4 File). Airorhynchy is positively allometric among colobinans (Pearson’s *r* = 0.75, p < 0.001), and *C. congoensis* falls above the regression line, more similar to *Colobus* than *Piliocolobus* of similar size (y = 0.55*x* – 1.15; R^2^ = 0.58; p < 0.001; Fig 5A). *Colobus congoensis* has a shortened petrous, more similar to *Colobus* or *Procolobus*, whereas *Piliocolobus* has a more elongated petrous (ANOVA F_(3, 134)_ = 18.35, p < 0.001; Tukey’s HSD *Piliocolobus* vs. *Colobus* and *Procolobus*, p < 0.01, see Table A in S4 File for all pairwise comparisons; Fig 5B). The difference in petrous shape between *Piliocolobus* and *C. congoensis* approaches significance, likely reflecting the small sample size (Tukey’s HSD p = 0.076; Table A in S4 File). Qualitatively, *C. congoensis* shares with *Colobus* a broadly incised posterior margin of the vomer that forms a “U” shape between the alae in a majority of specimens [32]; a relatively thin supraorbital torus, a mandibular corpus that deepens slightly, and a variably expanded gonion (Fig 4; Figs A-C in S4 File). Considering this combination of craniomandibular features, *C. congoensis* is consistently most similar to *Colobus* species relative to *Piliocolobus* or *Procolobus*.

In addition, *C. congoensis* lacks several qualitative cranial features characteristic of *Piliocolobus* and/or *Procolobus*, particularly the extensive sagittal and nuchal cresting seen in males of these taxa (Fig 4). Instead, *C. congoensis* lacks a sagittal crest in the lone male specimen, which exhibits widely spaced temporal lines and moderately developed nuchal cresting. Female *C. congoensis* also exhibit well developed, widely spaced temporal lines with weak to moderate nuchal cresting. Likewise, in the mandible, *C. congoensis* is distinguished from *Piliocolobus* and *Procolobus* by mandibular corpora lacking *prominentia laterales* and/or shallow mandibular fossae, and there is no median mental foramen perforating the mandibular symphyseal face (seen in *Procolobus*, specifically). Given these similarities with *Colobus* and the results of the phylogenetic analyses (below), we focused our further comparisons of *C. congoensis* within the genus *Colobus*.

#### 3.2.3 Comparisons to other *Colobus* species, with special reference to *C.* *satanas.*

**Fig 6:**
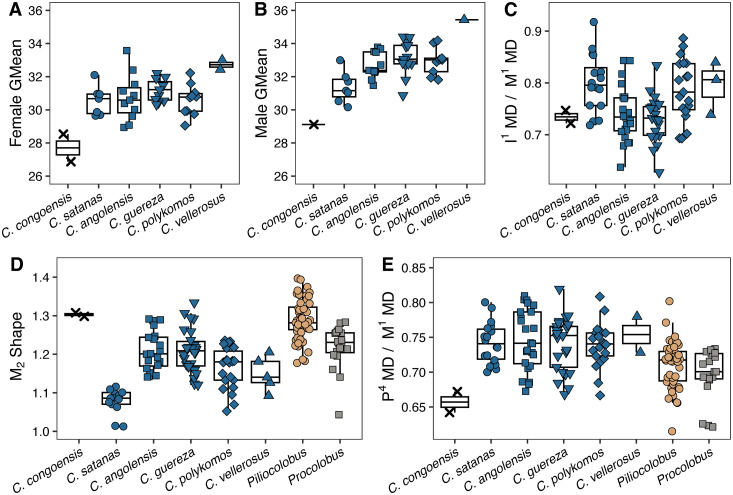
Craniodental indices differentiating *C. congoensis* and *C. satanas.* In both females **(A)** and males **(B)**, *C. congoensis* cranial size is smaller than all other species of *Colobus*. **(C)** *C. satanas* has relatively broader central incisors compared to other species of *Colobus*, which *C. congoensis* lacks. **(D)** In lower molar shape, *Colobus* has shorter molars than *Procolobus* and *Piliocolobus*, and within *Colobus*, *C. satanas* has uniquely mesiodistally shortened, buccolingually broad lower molars. *C. congoensis* is differentiated from *C. satanas* and other species of *Colobus* in exhibiting elongated lower molars. **(E)** *C. congoensis* lacks the enlarged P^4^ characteristic of *Colobus* relative to other African colobines. Skull indices are described in Table 2; MD = mesiodistal.

In life, *C. congoensis* differs from all other members of its genus by its striking piebald facial pattern, extensive white perianal patch, smaller body mass, and small cranium. Measurements of the available specimens suggest limited body mass sexual dimorphism in adults, although the available data on body weight includes a young adult male and two gravid females, likely skewing any body mass dimorphism ratio (Fig 6A & 6B; Table B in S1 File; S3 Table). Although additional body mass data will clarify the range of sexual size dimorphism, the available data indicate that *C. congoensis* is smaller in overall body mass than other *Colobus* species but exhibits moderate to low body mass and canine dimorphism within the range of other *Colobus* species, most similar to *C. satanas* (Fig 6A & 6B; Table B in S1 File; Text A in S4 File). S2 Table provides a comparison of selected features of the external morphology of *C. congoensis* with that of other species in the genus *Colobus*.

Among species of *Colobus*, *C. congoensis* exhibits notable similarities to *Colobus satanas* (Figs 2, 5). The nearly uniform black pelage covering the torso is similar in both species, in striking contrast to the white and black markings of all other *Colobus* species (Fig 2; Fig D in S4 File). Both *C. congoensis* and *C. satanas* have crenulated ear pinnae, markedly so in *C. satanas*. Prominent, long black hairs frame the forehead and sides of the face in both species*.* Fig 2 shows a number of external morphological features of *C. congoensis* and *C. satanas* as seen in life.

In the skull of *C. congoensis* and *C. satanas*, the frontal squama rises superiorly, and in lateral view, is continuous with the nasal profile unbroken by the glabella. The frontal of *C. congoensis* variably exhibits a slight bulging similar to the “saddle-shape” seen in *C. satanas* (*sensu* Groves [55]), although it is not as pronounced (Fig 4). Palate shape is allometric across all African colobines (Pearson’s *r* = 0.65; *p* < 0.001) but not within *Colobus* (Pearson’s *r* = 0.39; *p* < 0.001), and both *C. congoensis* and *C. satanas* have significantly shorter palates than most species of *Colobus* (ANOVA F_(5, 88)_ = 29.54; p < 0.001; Tukey’s HSD p < 0.05 for all pairwise comparisons with *C. congoensis* and *C. satanas* except *C. polykomos*, see also Table A in S4 File; Fig 5C). The mandibular corpus is shallow in both *C. congoensis* and *C. satanas* especially relative to *C. guereza* and *C. polykomos* (ANOVA F_(5, 76)_ = 7.39, p < 0.001; Tukey’s HSD p < 0.05, see also Table A in S4 File; Fig 5D). The male canine is relatively small for both *C. satanas* and the single male of *C. congoensis*, though this difference is not statistically supported in pairwise comparisons for *C. congoensis*, likely due to the small sample sizes (ANOVA F_(5, 38)_ = 2.61, p < 0.05; Fig 5E); *C. satanas* is significantly different from *C. angolensis* and *C. polykomos* (p < 0.05; Table A in S4 File). Though some of these craniodental comparisons between *C. congoensis* and other *Colobus* species are not statistically significant due to small sample sizes for *C. congoensis*, it consistently falls closest to the mean for *C. satanas* among living *Colobus* taxa in these distinctive features (Fig 5).

Although most similar to *C. satanas* among *Colobus* species, *C. congoensis* also exhibits a number of other features that are distinct from *C. satanas*, and in some cases, from all other species of *Colobus*. From field measurements and observations, *C. congoensis* is smaller in overall body mass and significantly smaller in cranial size compared to all other *Colobus* species, including *C. satanas* (combined sex ANOVA F_(5, 79)_ = 6.81, p < 0.001; Tukey’s HSD p < 0.001; see also Table A in S4 File; Fig 6A & 6B). In the pelage, the facial skin of *C. congoensis* has pale patches around the mouth and orbits, whereas in *C. satanas* this part of the face is entirely black (Fig 2; S2 Table). The mid-dorsum hair of *C. congoensis* is shorter on average (~5–12.5 cm) than that seen in *C. satanas*, which has longer mid-dorsum hair (12–16.5 cm; Fig D in S4 File). In *C. satanas*, a long tuft of hairs is present at the proximal base of the tail (extending ~15–18 cm distally from the base of the tail), whereas the hair on the rest of the tail is short (~1.5–3 cm). The tail of *C. congoensis* lacks a similar tuft at the base, and the hairs are only slightly longer at the base of the tail (~2–3 cm) than along the rest of the tail (~1–1.5 cm). *Colobus congoensis* has a distinct and extensive white perianal patch not found in *C. satanas*. In other *Colobus* species, any perianal white hair patches are typically more restricted to areas near and surrounding the callosities (S2 Table). Additional descriptions of *C. congoensis* pelage and comparisons to *C. satanas* are provided in Text A in S4 File.

Further comparisons of craniodental features corroborate the distinctiveness of *C. congoensis* from *C. satanas*, although small sample sizes again prevent statistical significance in some comparisons (Fig 6). Qualitatively, the frontal bulging in lateral view that is noticeably present in *C. satanas* (i.e., “saddle-shape” of [55]) is less pronounced in *C. congoensis* (Fig 4). *Colobus satanas* is distinct from other *Colobus* species in having enlarged upper central incisors and mesiodistally short, buccolingually broad lower molars, and *C. congoensis* lacks both of these features (Fig 6C-6D). In I^1^ breadth, *C. satanas* has significantly broader incisors than *C. angolensis* and *C. guereza* (ANOVA F_(5, 78)_ = 5.21; p < 0.001; Tukey’s HSD p < 0.01, see Table A in S4 File for all pairwise comparisons; Fig 6C). Although *C. congoensis* is not statistically significantly differentiated from *C. satanas*, it does exhibit relatively narrow incisors and falls at the bottom of the range of variation for *C. satanas* (Tukey’s HSD p = 0.56; Fig 6C). *Colobus* as a genus typically has shorter molars than *Procolobus* and *Piliocolobus* (ANOVA F_(3, 156)_ = 37.96, p < 0.001; Tukey’s HSD p < 0.05), and within *Colobus*, *C. satanas* has uniquely mesiodistally shortened, buccolingually broad lower molars (ANOVA F_(5, 82)_ = 16.82, p < 0.001; Tukey’s HSD p < 0.001 for all except *C. vellerosus*; Table A in S4 File). *Colobus congoensis* has elongated molars (p < 0.05 compared to *C. satanas*, *C. polykomos*, and *C. vellerosus*), and it is differentiated from *Colobus* as a genus (p < 0.05), resembling *Piliocolobus* in this index. In addition to molar shape, *C. congoensis* is differentiated from the rest of *Colobus* in exhibiting a small P^4^ relative to M^1^ length, more similar to that of *Procolobus* and *Piliocolobus* (ANOVA F_(3, 156)_ = 17.05, p < 0.001; Tukey’s HSD *Colobus* vs. all groups, p < 0.01, Table A in S4 File; Fig 6E). In molar shape and relative P^4^ size specifically, *C. congoensis* is more similar to *Piliocolobus* and *Procolobus* than other members of *Colobus*.

A multivariate assessment of these indices demonstrates the distinctiveness of *C. congoensis* relative to *C. satanas* and all other species of *Colobus* (see Tables B-D and Figs E & F in S4 File). Phenetic distances between *C. congoensis* and *C. satanas* are greater than between any other pair of *Colobus* species (Fig E and Table C in S4 File) or pair of subspecies (Fig F and Table D in S4 File).

### 3.3 Genetics

**Fig 7:**
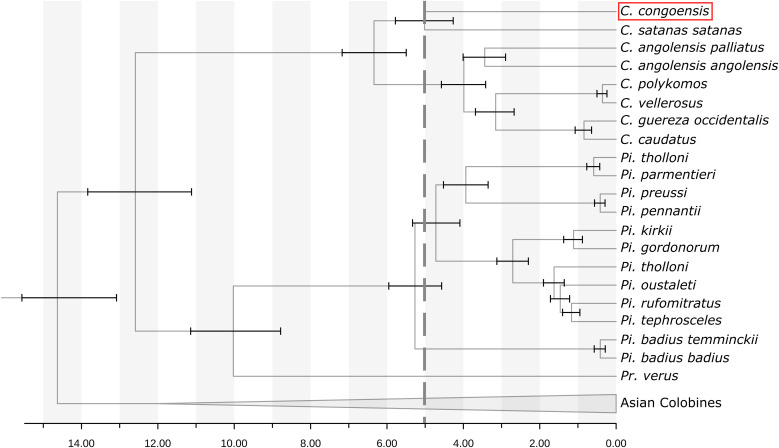
Estimated divergence dates of African colobines using a fossil calibration. MCMCTree divergence date estimates using available mitochondrial data and a fossil-calibrated prior from de Vries and Beck [43]. The error bars on the nodes represent 95% highest posterior density intervals. The dashed gray line highlights the inferred divergence date between *C. congoensis* and *C. satanas*. *Colobus congoensis* is highlighted by a red rectangle.

Of the three *C. congoensis* mitochondrial DNA sequences that were generated in this study, two had unique haplotypes and were included in the phylogenetic analyses. The final alignment consisted of 46 African colobine samples, including representatives of the six currently recognized *Colobus* species, 10 species of *Piliocolobus*, and *Procolobus verus*, together with seven samples from Asian colobines (S1 Table). Our phylogenetic analyses of mitochondrial DNA sequences recover the *C. congoensis* samples as a monophyletic clade that is sister to *C. satanas*; together, the *C. congoensis–C. satanas* clade forms the sister to all other *Colobus* species (Fig 7; Fig A in S5 File). Mitochondrial sequences from two *C. angolensis* individuals from the buffer zone of Lomami National Park form a monophyletic clade that is the sister to a *C. angolensis* clade that includes one individual from Tanzania and two of unknown provenance (Fig A in S5 File).

Using a calibration point on the African*–*Asian colobine split from a recently published set of curated primate fossil calibrations [43], the split between *C. congoensis* and *C. satanas* is estimated at 5.0 million years ago (Ma; 95% highest posterior density [HPD] = 4.3–5.8 Ma; Fig 7, Table A in S5 File). The divergence of the sympatric Lomami *C. angolensis angolensis* population from other *C. angolensis* populations is estimated at 3.4 Ma (95% HPD = 2.9–4.0 Ma; Fig 7, Table A in S5 File). When using the estimated divergence date from Perelman et al. [45] as the calibration on the Colobinae node, the split between *C. congoensis* and *C. satanas* is estimated at 4.1 Ma (95% HPD = 3.6–4.7 Ma), and the split between the Lomami *C. angolensis angolensis* population and other *C. angolensis* populations is estimated at 2.8 Ma (95% HPD = 2.4–3.3 Ma; Fig B and Table A in S5 File).

### 3.4 Vocalizations

Roaring calls were heard at all times of the day, but particularly in the morning. The pale coloration around the mouth produces a distinctive visual display in roaring *C. congoensis* (S1 Video). Multiple individuals were observed calling together on several occasions. In one observation of a social group (n = ~10 individuals), we heard an intense bout of rapidly repeated roars during an attack by a crowned eagle (*Stephanoaetus coronatus*). These observations show that *C. congoensis*, like other *Colobus* species, roars in varied situations, including when under threat from potential predators [46,56].

We analyzed *C. congoensis* roars from three audio files, all of which included more than one individual calling during the event. In the field, *C. congoensis* roars are similar to those of sympatric *C. angolensis* but can be distinguished by the rapid pulse rate of roars and a distinctive snort between roar sequences. Snorts by *C. angolensis* are often produced alone and independent of roaring [46]. A video recording of roaring *C. congoensis* is provided in S1 Video.

#### 3.4.1 Comparisons within the genus *Colobus.*

Oates and Trocco [48] and Oates et al. [47] compared features of roars of five *Colobus* species: *C. guereza*, *C. polykomos*, *C. satanas*, *C. angolensis*, and *C. vellerosus*. They identified three characteristics within roar phrases that could be used to group species: pulse rate (pulses per second), peak fundamental frequency (Hz), and the presence of frequency modulation. We compared *C. congoensis* roars with these five species plus *C. caudatus* (reported as *C. guereza caudatus* in [47,48]). *Colobus guereza*, *C. caudatus*, and *C. vellerosus* roars had a slow pulse rate and low fundamental frequency. *Colobus angolensis* and *C. polykomos* roars had an intermediate pulse rate and intermediate or high fundamental frequency. *Colobus satanas* roars had a rapid pulse rate and high fundamental frequency with frequency modulation. *Colobus congoensis* roars are structurally closest to those of *C. satanas* with rapid pulse rate, high fundamental frequency, and frequency modulation across the call (Table 4).

**Table 4:** Comparative summary of roar features for *Colobus* species.

Parameter	*C. angolensis*	*C. polykomos*	*C. guereza*	*C. caudatus*	*C. vellerosus*	*C. satanas*	*C. congoensis* ^3^
Peak Fundamental Frequency (Hz)^1^	x̄ 614–671 (510–723)	x̄ 672–710 (581–771)	x̄ 481–669 (439–700)	x̄ 621–671 (593–700)	x̄ 525–554 (454–593)	x̄ 690–780 (617–818)	x̄ 733 (517–891)
Pulse Rate (pulses/second)^1^	x̄ 33.8–42.9 (29.2–57.5)	x̄ 27.6–34.5 (24.8–39.8)	x̄ 17.1–22.9 (13.3–25.1)	x̄ 21.5–22.5 (13.8–24.6)	x̄ 20.6–22.7 (16.3–26.0)	x̄ 55.0–69.3 (46.0–81.5)	x̄ 56.4 (45–60)
Frequency Modulation^2^	No	No	No	No	No	Yes	Yes

1 Reported values by Oates et al. [47] for all listed species except *C. congoensis*. Oates et al. [47] reported the mean and range for each individual in their sample. We report the range of means and the range of values for each species in their study.

2 Reported presence or absence of frequency modulation by Oates and Trocco [48] for all listed species except *C. congoensis*.

3 Results from this study for *C. congoensis*. Sample size includes 245 roars from 3 audio files.

#### 3.4.2 Comparison of *C. satanas* and *C. congoensis* roars.

**Fig 8:**
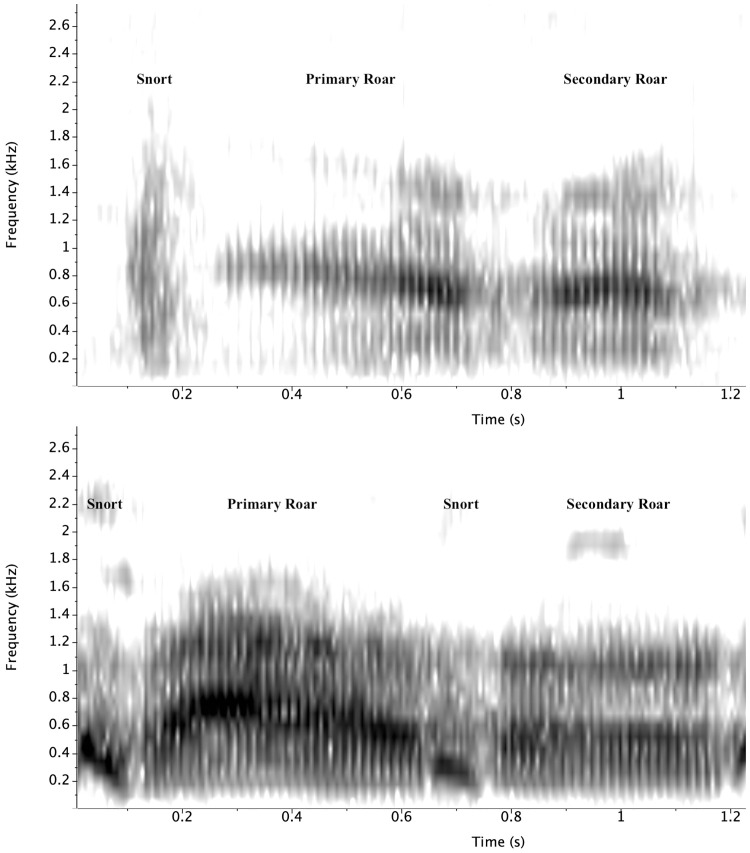
Spectrogram of *C. congoensis* and *C. satanas.* Spectrogram of *C. congoensis* (top) and *C. satanas* (bottom) roars. Primary roars in both species are preceded by a snort and show frequency modulation. Secondary roars show little to no frequency modulation and are preceded by snorts in *C. satanas*, but not in *C. congoensis*. Audio files that include the calls depicted in the spectrograms can be found in S1 Audio (*C. congoensis*) and S2 Audio (*C. satanas*).

For our comparative analysis of *C. congoensis* and *C. satanas* roars, we used 86 *C. congoensis* roar phrases from one audio file with at least two individuals calling. We extracted 101 *C. satanas* roar phrases from two audio files with at least three individuals calling. Roars by *C. congoensis* and *C. satanas* have two dominant frequency bands and discrete pulses within each phrase. Two roar phrase variants were visually identified using spectrograms: a pronounced roar considered to be a ‘primary roar’ and a less pronounced roar considered to be a ‘secondary roar’ (Fig 8). Primary roars were longer in duration and exhibited frequency modulation across the call. Secondary roars were shorter in duration with less frequency modulation than the primary roar preceding it. The intensity of frequency modulation varied among primary phrases in both species. Primary roars were either isolated or started a sequence of phrases that alternated or ended with a secondary roar. In several instances, a primary roar by *C. congoensis* was followed by two secondary roars. Primary roars for both species were almost always preceded by a snort vocalization. Secondary roars in *C. satanas* were separated from primary roars by a snort, whereas *C. congoensis* did not snort between primary and secondary roars. Audio recordings of roaring *C. congoensis* and *C. satanas* are provided in S1 Audio and S2 Audio, respectively.

*Colobus congoensis* roars were higher in frequency on several acoustic features and had a larger formant dispersion between dominant frequency bands compared to *C. satanas* roars. Primary roars of *C. congoensis* consistently exhibited a negative slope, whereas those of *C. satanas* displayed an arched contour of varying intensity in the fundamental frequency. However, secondary roars for *C. congoensis* and *C. satanas* did not differ in their slope. Primary roars were similar in duration and rise time, but in secondary roars, duration and rise time were shorter in *C. congoensis* compared to *C. satanas*. Pulse rate was similar for both species across both call variants. Using principal component analysis (PCA), both call variants formed species-specific clusters for *C. congoensis* and *C. satanas* (Figs A & B in S2 File, and Tables A-E in S2 File). Summary statistics for acoustic features of roar calls are reported in Table A in S2 File.

### 3.5 Distribution, ecology and behavior

The known range of *Colobus congoensis* is limited to an area of 1,700 km^2^ in the provinces of Tshopo and Maniema in east-central DRC. Its range extends from the right bank of the Lomami River, east into the adjacent Lualaba basin to the Lilo River, a tributary of the Congo (Lualaba) River. The northern known limit is at approximately 1°20’ South Latitude. The southern limit is approximately 2° South Latitude (Fig 1). *Colobus congensis* is not known from the west bank of the Lomami River despite the occurrence of apparently suitable habitat. Elevations in the range area vary from 390 to 500 meters above sea level (a.s.l.), with observations of the species recorded from 406 to 490 meters a.s.l. Annual rainfall recorded at two sites bordering the north and south of the *C. congoensis* range averaged 1,870 mm and 1,720 mm, respectively. Rainfall seasonality is bimodal: October through December and March through April are the wettest months with monthly rainfall averaging 201–269 mm. June and July are the driest months, with monthly rainfall averages of 34–111 mm.

**Fig 9:**
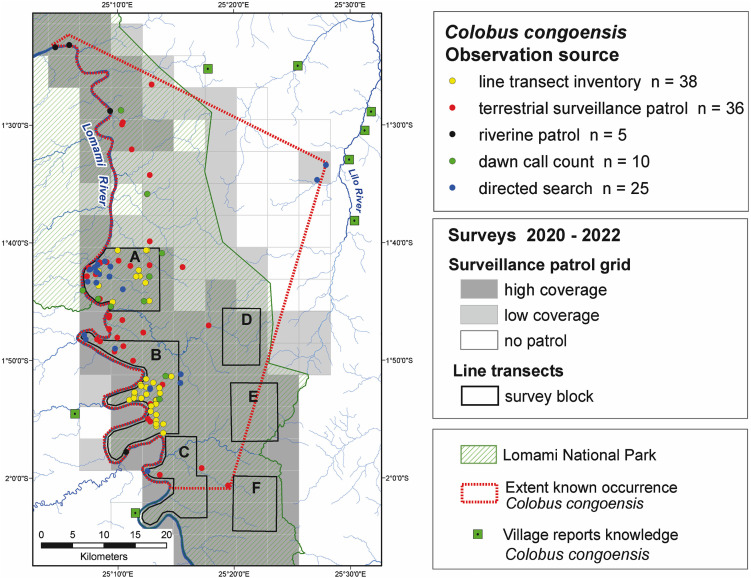
Observations of *Colobus congoensis*, 2018–2022. Following its discovery in November 2018, an additional eight observations of *C. congoensis* were made through 2019. Beginning in 2020, surveillance patrol coverage, directed searches, and dawn call surveys were increased in the range area, and line transect surveys initiated. One hundred five of 114 observations were recorded from 2020 through 2022. Surveillance patrol surveys were deployed on a 5 × 5 km grid. Grids with high coverage had at least 3 patrols per year and an accumulation of 30 km of patrol track. Low coverage patrol grids had lower annual and/or total patrol track coverage. Line transect coverage for survey blocks A–F is given in Table 5. Villages reporting knowledge of *C. congoensis* are restricted to the buffer zone bordering the Lomami National Park.

*Colobus congoensis* is currently known from 114 detections made between 2018 and 2022 (Fig 9, S4 Table). *Colobus congoensis* were observed visually on 89 encounters. During mixed-species associations, observers estimated the number of *C. congoensis* in 62 of the visual encounters. Twenty-five detections were by vocalizations only or by vocalization with little visual contact.

**Table 5:** Line transect inventory results.

Survey block	Block area (km^2^)	Predominant habitats	Total transect distance (km)^1^	Number of groups detected and encounter rate (number of groups/km transect)	
				*C. congoensis*	All primates
A	104	Pediment forest	335	11 (0.033)	77 (0.230)
B	129	Pediment forest	656	27 (0.041)	532 (0.811)
C	88	Low relief forest	94	None	33 (0.351)
D	53	*Sende* mosaic	51	None	32 (0.632)
E	68	*Sende* mosaic	81	None	18 (0.223)
F	59	*Sende* mosaic	68	None	17 (0.250)
**Total**	**501**		**1285**	**38 (0.030)**	**709 (0.552)**

^1^Total accumulated transect distances summed across all observers in each block.

*Colobus congoensis* is an uncommonly encountered monkey within its range (Fig 9). Eight other primate species were documented in the range area, including seven monkey species and the Bonobo, *Pan paniscus* (S5 Table). Detection rates within the range area (detections / survey effort) for *C. congoensis* were evaluated for all survey methods, except for 25 detections made on directed searches. Between 2020 and 2022, 3,063 kilometers of terrestrial surveillance patrols were deployed within the *C. congoensis* range. Twenty-nine of a total of 36 *C. congoensis* detections by surveillance patrols were made during this period, for an overall encounter rate of 0.01 detections per km of patrol recce. Surveillance patrol detections were recorded in just 20 of 76 patrol grid cells (5 × 5 km) covered by patrols within the *C. congoensis* range. *Colobus congoensis* detection rates were low on riverine patrols; 790 km of riverine coverage from 2020 through 2022 yielded only 5 encounters of *C. congoensis.* Line transect inventories, totaling 1,285 km across all 6 survey blocks, yielded a total of 38 *C. congoensis* observations out of a total of 709 detections of all primates (Table 5). *Colobus congoensis* was detected during transect inventories only in survey blocks A (11 detections) and B (27 detections), comprising 0.033 and 0.041 detections per km of transect for the two blocks, respectively, and representing 5.4% of all primate detections (Table 5). Dawn primate call surveys yielded patterns of *C. congoensis* detection rate and distribution similar to those produced by transect inventories. *Colobus congoensis* were recorded on only 10 of 351 call surveys within the species’ range over the 3-year period 2020–2022 (Fig A in S3 File).

*Colobus congoensis* are concentrated toward the western limits of the known range, with 104 of 114 detections recorded in pediment forest within 10 km of the Lomami River. Within this zone, observations of *C. congoensis* are further concentrated in the vicinity of four of the six major meanders constituting the dominant feature of the Lomami River’s course in the Courbure Sector of the Lomami National Park. East of this band, and stretching into the Congo (Lualaba) Basin, the landscape gives way to a mosaic of upland and *sende* forests. *Colobus congoensis* were reported from only two locations in the *sende* zone; both were islands of upland forest west of the Lilo River that are not subject to seasonal inundation.

Observed group size of *C. congoensis* ranged from 1 to 20 individuals, with an average of 6.2 and a median size of 5.5 individuals. *Colobus congoensis* shows a consistent tendency to move in mixed-species groups of primates. Of 62 observations where group composition was determined, *C. congoensis* was reported in a polyspecific association on 45 observations, with group compositions ranging from 2 to 6 species. Two other colobines present in the landscape were recorded in mixed groups with *C. congoensis*: *C. angolensis* was observed in association with *C. congoensis* five times and *Piliocolobus parmentieri* was observed once. Adult *C. congoensis* with young were observed in February, April, July, August, and September. In all cases, the young animals had the full black coloration and piebald facial pattern of adults. *Colobus congoensis* were observed primarily in the canopy or middle level of mature forest. Two exceptions were reported. In one event, two *C. congoensis* of a monospecific group of 5 individuals descended to the ground briefly before moving back up into the middle level. In the second event, a group of approximately 10 *C. congoensis* under attack from a crowned eagle (*Stephanoaetus coronatus*) moved from the canopy to the understory.

## 4 Discussion

### 4.1 The place of *C. congoensis* in the radiation of the genus *Colobus*

*Colobus congoensis* represents the sister taxon to the Black Colobus (*C. satanas*), long recognized to be an unusual member of the genus characterized by its all-black pelage, unique cranial morphology, and dietary ecology [48,55,57,58]. Our description of a new species most closely related to *C. satanas* thus represents a significant expansion of known *Colobus* diversity, with implications for understanding the evolution of the genus and of African colobines, more broadly. Our molecular analysis is consistent with previous phylogenies in positioning *C. satanas* (here, together with *C. congoensis*) as sister to a monophyletic clade of black-and-white *Colobus* species (*C. guereza*, *C. angolensis*, *C. polykomos*, *C. vellerosus*, and *C. caudatus*) [19,32,35,36,59].

Our fossil calibrated mitochondrial clock analysis estimates the most recent common ancestor of *C. congoensis* and *C. satanas* existed ~4.27–5.78 Ma. In an additional analysis that used the same secondary calibration point as Roos and Zinner [36], we obtained younger divergence estimates by approximately 1 million years (3.44–4.73 Ma). This difference is likely due to limitations of applying molecular dating methods to small mitochondrial datasets and differences in calibration approaches. Future research on comparative whole genome data (nuclear and mitochondrial) will provide more accurate divergence date estimates for the *Colobus* clade [e.g., 60,61].

A Pliocene age split between the *C. congoensis–C. satanas* lineages represents the deepest split between sister species within the genus *Colobus*, similar in age to the oldest species-group divergences within other speciose cercopithecid genera (i.e., *Macaca* and *Cercopithecus*) [59,61]. This divergence is additionally substantially older than between any subspecies among African colobines (Fig 7). The antiquity of this split, combined with distinct pelage and craniodental features, indicates that *C. congoensis* is an independently evolving lineage that deserves to be recognized as a novel taxon apart from its phylogenetically closest relative, *C. satanas*, at the species level [62]. Under a phylogenetic species concept [63], the specific status of *C. congoensis* is corroborated by several unique, diagnosable features relative to both its sister species, *C. satanas*, and all other species of *Colobus* (Figs 5 & 6, Figs E & F and Tables C & D in S4 File; see also formal diagnosis above and summary below). Structural analysis of loud call vocalizations identifies similarities between *C. congoensis* and *C. satanas* that distinguish them from all other *Colobus* species [47,48] but also highlights distinctive features that further separate *C. congoensis* in vocalization sequence, frequency modulation, and formant dispersion. Morphological similarities shared by *C. congoensis* and *C. satanas* that are not present in other *Colobus* species include the all-black pelage and the unusual saddle-shaped frontal bones. *Colobus congoensis*, however, is distinguished by its striking piebald facial skin markings; the white perianal patch; unique aspects of its dental morphology in relative size of the upper incisor, premolar proportions, and molar shape; and its overall small body size and cranial size. Multivariate analyses of phenetic distances also confirm the morphological distinctiveness of *C. congoensis* relative to *C. satanas* and all other *Colobus* species (Figs E & F and Tables C & D in S4 File).

For many aspects of its external appearance, *C. congoensis* is reminiscent of Asian colobine genera such as *Trachypithecus* and *Presbytis*, particularly in the light colored skin around the mouth, eye rings, and the presence of a crest/crown on the top of the head, raising the question of whether *C. congoensis* retains some pelage features from the common ancestor with Asian colobines. Similarities in external features to *Trachypithecus* and *Presbytis* have also been noted for *Procolobus verus*, which is the sister group to *Piliocolobus* [64]. These similarities suggest that both *Piliocolobus* and the other *Colobus* species are independently more derived in external appearance compared to *Procolobus* and the *C. satanas*–*C. congoensis* clade. Derived features in *Colobus* include the striking black and white coloration and elaborate manes, capes, and tail tufts seen in many members of the genus (S2 Table) [58,65,66].

Dental differences between *C. congoensis* and its congeners, particularly *C. satanas*, may also suggest a distinctive dietary ecology. *Colobus* species are characterized by mesiodistally shorter molars, greater basal flare, relatively larger premolars, and reduced canine base area dimorphism relative to *Piliocolobus*, and these features have been related to a greater degree of seed predation and mature leaf consumption within the genus [31,67–72]. Even relative to other *Colobus* species, *C. satanas* is highly granivorous, with seeds making up half or more of its diet [67,71]. The enlarged upper incisors, particularly mesiodistally shortened and buccolingually broad molars, and higher basal flare of *C. satanas* have been linked to its extensive seed predation [68,69,72]. On the other hand, *C. congoensis* lacks these distinctive dental features: it has small incisors, smaller premolars, and mesiodistally elongated molars. In these latter two features, it is also distinguished from all other species of *Colobus* and is more similar to *Piliocolobus*. This suggests that *C. congoensis* is likely not as granivorous as *C. satanas* and may rely on seeds even less than all other species of *Colobus*, with a dietary profile more similar to *Piliocolobus* species.

### 4.2 Habitat specialization in *C. congoensis*

*Colobus congoensis* is limited to upland forests, and in particular those on pediments with deep clay soils. The species’ occurrence outside of this habitat is highly localized. Upland clay substrates support some of the most diverse forests in Lomami National Park, with significantly higher tree species diversity than in forests on upland white sand soils or on seasonally inundated substrates [73,74]. This suggests that *C. congoensis* may be limited by habitat quality, and that nutrient poor and/or less diverse forests, including those occurring on white sand substrates, are not suitable habitats.

**Fig 10:**
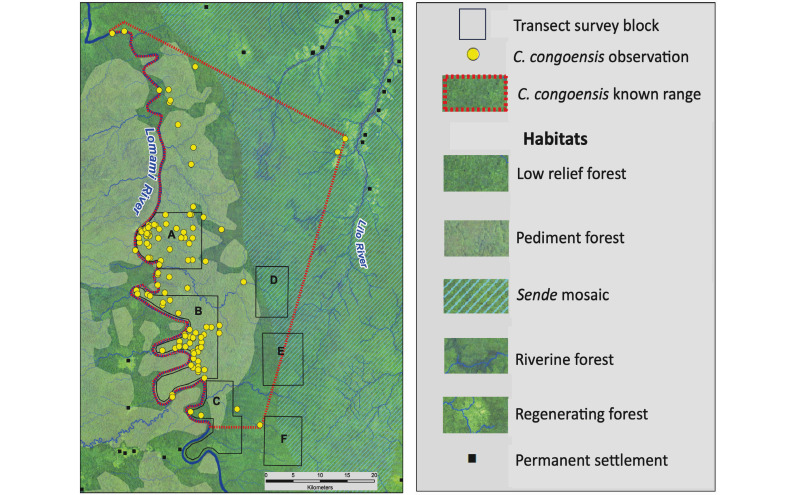
*Colobus congoensis* distribution and habitat. *Colobus congoensis* observations (2018–2022) shown relative to habitat classes (see Table 3 for habitat descriptions) within the known range.

At a finer geographic scale, the highly variable spatial distribution of canopy tree species in the terra firme, clay-substrate forest [74] suggests that local floristic composition may be further shaping the localized distribution of *C. congoensis.* Repeated observations of *C. congoensis* in the vicinity of the same location suggest that *C. congoensis* groups resample selected sites over time. *Colobus congoensis* is rare in the *sende* zone, which occurs at the eastern limits of the species’ range. We recorded only two occurrences in this zone, both on well drained islands of sandy clay where local informants led us to monkeys that they reported seeing regularly. Both these areas were also used on a temporary basis for shifting cultivation by people based in settlements east of the Lilo River (Fig 10).

Fragmented or geographically restricted distributions are not uncommon among primates, and are shaped by factors such as habitat requirements, biogeographic barriers, and faunal community composition. Among African colobines, for instance, populations of *Piliocolobus oustaleti* persist in small islands of gallery forests isolated in savannas on the escarpment overlooking Lake Albert in the DRC [75]. Similarly, in Lomami National Park, *Piliocolobus parmentieri* has a patchy distribution in the Lomami-Lualaba interfluve that appears to be associated with selection for islands of suitable habitat. Like *P. parmentieri*, the distribution of *C*. *congoensis* follows a comparable pattern in its rarity and localized distribution, suggesting that habitat specialization is most likely driving the fragmented distributions of these taxa.

### 4.3 Biogeography and range restriction of *C. congoensis*

*Colobus congoensis* is limited to an unusually small geographic range currently estimated at 1,700 km^2^ (Fig 1), while most other known species of the genus have more extensive ranges exceeding 60,000 km^2^ [76]. The range of *C. congoensis* is notably separated from its closest phylogenetic relative, *C. satanas* in western central Africa, by at least 1,200 km. Both the limited distribution of *C. congoensis* and the geographic separation from its sister species raise questions about the historical biogeography of the genus *Colobus* and local biogeographic factors limiting its range.

A west African origin for both the *Colobus* and *Procolobus–Piliocolobus* clades, and by extension, African colobines as a group, has been proposed on the basis of the deep divergences between *C. satanas* and its congeners and between *Procolobus* and *Piliocolobus* [77,78]. However, *C. satanas* does not represent the westernmost extension of the *Colobus* range (Fig 1b), and the position of *C. congoensis* as sister to *C. satanas* in the Congo Basin further complicates this picture. The distribution of *Colobus* species does not follow a straightforward west-east phylogeographic pattern, and thus the geographic origins of the genus are difficult to discern. Complicated west-east biogeographic dispersal scenarios have also been hypothesized for several other African catarrhine primates, including *Cercocebus* mangabeys [79], chimpanzees [80,81], some guenon clades [82–84], and notably, red colobus monkeys [35,85]. Identifying the origins of the *C. satanas–C. congoensis* clade as either within or outside the Congo Basin remains obscure. Regardless, the relative evolutionary isolation of *C. congoensis* may suggest it is a relictual member of a previously more diverse radiation within the Congo Basin, possibly confined to its small current range by competitive exclusion (e.g., by *Piliocolobus* species) and/or by habitat specialization.

Molecular estimates for the origins of several colobinan lineages date to the late Miocene or early Pliocene [35,45,59,61]. Our mitochondrial clock analyses estimate the split between *C. satanas* and *C. congoensis* at ~4.1–5.0 Ma. The antiquity of this split indicates that the divergence of the two taxa was unlikely to be directly due to Pleistocene glacial cycles and the formation and expansion of forest refugia, as proposed for other speciose cercopithecid radiations such as the guenons (tribe Cercopithecini), *Cercocebus* mangabeys, or *Piliocolobus* [35,48,77,79,85]. Rather, riverine dynamics and the evolution of central African geomorphology likely played a role in the differentiation of *C. satanas* and *C. congoensis* and the current restriction of the *C. congoensis* range [86,87].

While the current course of the Congo River dates to the end of the Eocene or beginning of the Oligocene, 30–40 million years ago [88], the configuration of the Congo’s central basin tributary watersheds developed more recently in the late Pliocene through the Quaternary [89]. North-south oriented drainages with sources on the periphery of the Central Basin, such as the Lomami River, are thought to have developed over the past 2 million years [89]. The appearance of dissected pediments, including those occupied by *C. congoensis*, are dated to the late Pliocene [52, Plate 2]. Collectively, this suggests that the modern configuration of the *C. congoensis* range may have been in place by the late Pliocene or early Pleistocene, postdating the estimated age of the split between *C. satanas* and *C. congoensis*.

The rivers that bound the *C. congoensis* range to the west and east appear to represent biogeographic barriers for the species through different mechanisms. The Lomami River, which defines the western boundary of the *C. congoensis* range, borders the species and subspecies ranges of several other primates in the Lomami National Park and buffer zone region (S5 Table). With an average width of over 200 meters, the Lomami River appears to represent a significant barrier to dispersal. At the eastern limit of the *C. congoensis* range, the Lilo River is less than 50 meters wide, and in some places is covered by canopy traversable by arboreal primates. However, the Lilo River and all other major tributaries within the Lomami-Lualaba interfluve in the *C. congoensis* range have “elbows,” right-angle changes of course that are characteristic of captured or “beheaded” rivers (Fig 1) [90]. All these rivers are also characterized by depositions of white sands and seasonal inundations in regions that were occupied by the former course of the captured flowage. Nutrient-poor, white-sand soils and their associated vegetation have been recognized as ecological barriers for a number of plant and animal taxa in Amazonia, and in some cases have been demonstrated to promote allopatric speciation [91–93]. Such habitats have not previously been identified as geographic barriers limiting primate ranges in Africa, as seems to be the case for *C. congoensis*. The effect of these nutrient-poor habitats in the Lomami-Lualaba interfluve in defining the range limits of *C. congoensis* would be better understood with an assessment of the species’ diet, as the relationship between nutrient limitation and primate distribution and abundance has been shown to be mediated through diet choice and food availability [67,94–96].

### 4.4 Conservation status

We propose a provisionary IUCN Red List classification for *C. congoensis* of Endangered (EN). *Colobus congoensis* is a rare, geographically restricted monkey that is not well known even by local communities in the vicinity of its range. Mean group size and encounter rates on surveys are low, and *C. congoensis* sightings are highly localized and mostly limited to a narrow area in the vicinity of the Lomami River. According to local informants, it appears that the species was not specifically targeted by hunters in the past, nor has its range been reduced in living memory. Most of the known locations of *C. congoensis* occur within the protected Lomami National Park.

Butynski and de Jong [97] list seven predictors of heightened risk of extinction in colobines, of which three are pertinent to *C. congoensis* at present: dependence on high canopy, old growth forest, and a body size greater than 5 kg. Among the remaining predictors, habitat loss, human population growth, and increased hunting in the range can be projected as threats to this species over the upcoming decades. Human population growth in DRC is estimated at 3.2% annually, one of the most rapidly growing national populations on the continent [98]. Population growth is one of the most important drivers of forest loss. Over 3,300 km^2^ of primary forest is projected to have been lost in the DRC between 2015 and 2025 [99]. Loss of primary forest cover in Tshopo Province, which includes a significant portion of the *C. congoensis* range, is estimated at 4% annually [100].

The *C. congoensis* range in the buffer zone of Lomami National Park is located in an area where the local economy is dependent on hunting, fishing, and shifting cultivation. Furthermore, the area is characterized by extensive seasonally inundated forests, reducing the availability of terra firme islands within the sector, preferred by both *C. congoensis* and humans. Expansions of human populations from the current areas of settlement are ongoing, with at least 15 new villages added within and adjacent to the range of *C. congoensis* in the Lomami National Park buffer zone between 2015 and 2023 (Lomami National Park, unpublished data). If these demographic and land use changes continue, we anticipate significant reductions of *C. congoensis* populations and loss of habitat in the buffer zone over the upcoming decades.

Effective protection of Lomami National Park emerges as the most important requirement for the conservation of *C. congoensis.* The discovery of *C. congoensis* was preceded by the discoveries of two previously unknown or little known cercopithecids in the Lomami National Park and buffer zone: *Cercopithecus lomamiensis* [28] and *Chlorocebus dryas* [101–103]. The latter was previously known from a single small range area over 400 km to the west.

The known primate fauna of the Lomami National Park and its buffer zone totals 15 species, including 8 species endemic or near endemic to the DRC (S5 Table). Although many of these primate species are also found outside of the TL2 interfluves, the discovery of *C. congoensis* along with the novel species *Ce. lomamiensis* and the very poorly known *Ch. dryas* points to a gap in scientific knowledge of the biodiversity in this region. These discoveries confirm the exceptional importance of the TL2 landscape for primate biogeography and evolution in Congo’s Central Basin. The Lomami National Park is one of the most important protected areas for primate conservation in Central Africa.

## Supporting information

S1 File Skeletal metrics of *Colobus congoensis* and comparative sample. (XLSX)

S2 File Supplementary vocalization analyses of *Colobus congoensis.* (DOCX)

S3 File Extended description of field survey methods. (DOCX)

S4 File Extended descriptions and comparisons of *Colobus congoensis* skins and skulls, and supplementary morphometric analyses. (DOCX)

S5 File Supplementary phylogenetic trees and divergence date estimates. (DOCX)

S1 Matrix Mitochondrial DNA sequence alignment. (NEXUS)

S1 Text Questionnaire: Inclusivity in global research. (PDF)

S1 Table Samples included in phylogenetic analyses. (XLSX)

S2 Table Comparison of selected external morphological characters in adult *Colobus.* (DOCX)

S3 Table Field measures of *Colobus congoensis* specimens. (XLSX)

S4 Table Field observations of *Colobus congoensis.* (XLSX)

S5 Table Primates of the Tshuapa-Lomami-Lualaba (TL2) interfluves. (DOCX)

S1 Video *Colobus congoensis* recorded on a video while vocalizing. (MP4)

S1 Audio *Colobus congoensis* vocalizations. (WAV)

S2 Audio *Colobus satanas* vocalizations. (WAV)
