# Supplementary material for: Likweli: A remarkable new species of Colobus monkey from the Lomami National Park, Democratic Republic of Congo
Source: PLoS One. 2026 Jul 15;21(7):e0349857. doi: 10.1371/journal.pone.0349857 (PMC13372154; doi:10.1371/journal.pone.0349857)
Supplement: S2 File — (DOCX) [file pone.0349857.s002.docx]

**Supplementary vocalization analyses of *Colobus congoensis***

**Table A: Summary statistics for primary and secondary roars for *C. congoensis* and *C. satanas***

|  | ***C. congoensis***  **Primary roars**  **(n=62)** | | ***C. satanas***  **Primary roars**  **(n=68)** | | ***C. congoensis***  **Secondary roars**  **(n=23)** | | ***C. satanas***  **Secondary roars**  **(n=28)** | |
| --- | --- | --- | --- | --- | --- | --- | --- | --- |
| **Acoustic Feature** | **Mean±SD** | **Range** | **Mean±SD** | **Range** | **Mean±SD** | **Range** | **Mean±SD** | **Range** |
| **TOTAL CALL** | | | | | | | | |
| Maximum frequency  (Hz) | 701.57 ± 54.80 | 559.86- 775.20 | 669.20 ± 44.78 | 581.40- 753.66 | 640.38 ± 82.44 | 516.80- 775.20 | 575.24 ± 113.17 | 409.13- 1098.20 |
| 25% frequency  (Hz) | 622.38 ± 69.61 | 473.73- 732.13 | 605.01 ± 49.74 | 409.13- 710.60 | 565.48 ± 93.01 | 430.66- 689.06 | 476.04 ± 49.15 | 387.60- 559.86 |
| 50% frequency (Hz) | 693.92 ± 52.95 | 559.86- 818.26 | 678.83 ± 49.97 | 581.40- 890.62 | 634.76 ± 79.22 | 516.80- 775.20 | 567.55 ± 59.25 | 452.20- 753.66 |
| 75% frequency (Hz) | 768.25 ± 96.52 | 646.00- 1335.10 | 784.64 ± 162.33 | 667.53- 1453.10 | 685.32 ± 79.95 | 559.86- 818.26 | 709.83 ± 166.64 | 538.33- 1098.20 |
| Rise time (s) | 0.23 ±  0.05 | 0.13- 0.35 | 0.25 ±  0.08 | 0.07-  0.51 | 0.12 ± 0.05 | 0.05- 0.24 | 0.16 ± 0.07 | 0.06-  0.34 |
| **FUNDAMENTAL FREQUENCY BAND** | | | | | | | | |
| Duration (s) | 0.45 ±  0.09 | 0.26- 0.70 | 0.49 ± 0.16 | 0.14- 1.02 | 0.23 ± 0.10 | 0.10- 0.47 | 0.32 ± 0.13 | 0.12-  0.67 |
| Lower frequency limit (Hz) | 86.84 ± 26.16 | 26.76- 168.59 | 95.87 ± 30.63 | 0.00-  155.90 | 89.37 ± 30.29 | 29.32- 198.02 | 105.90 ± 14.94 | 64.72-  132.37 |
| Upper frequency limit (Hz) | 1050.90 ± 139.71 | 842.93- 1758.10 | 972.73 ± 72.25 | 832.46- 1156.00 | 950.21 ± 105.86 | 789.41- 1142.60 | 837.46 ± 71.98 | 685.39- 944.24 |
| 25% frequency (Hz) | 609.18 ± 73.92 | 430.66- 732.13 | 595.13 ± 48.42 | 409.13 710.60 | 561.74 ± 93.17 | 430.66- 689.06 | 452.97 ± 49.55 | 387.60- 559.86 |
| 50% frequency (Hz) | 684.89 ± 55.53 | 559.86- 775.20 | 659.70 ± 35.41 | 581.40- 750.00 | 631.02 ± 74.15 | 516.80- 732.13 | 539.10 ± 44.05 | 452.20- 602.93 |
| 75% frequency (Hz) | 740.46 ± 45.36 | 646.00- 818.26 | 711.91 ± 32.80 | 624.46- 775.20 | 675.96 ± 75.08 | 559.86- 775.20 | 592.93 ± 42.86 | 516.80- 667.53 |
| Maximum frequency (Hz) | 701.57 ± 54.80 | 559.86- 775.20 | 669.20 ± 44.78 | 581.40- 753.66 | 640.38 ± 82.44 | 516.80- 775.20 | 556.02 ± 47.99 | 409.13- 646.00 |
| Interquartile range (Hz) | 131.28 ± 60.62 | 43.07- 301.47 | 116.78 ± 42.25 | 43.07- 279.93 | 114.22 ± 35.82 | 43.07- 172.27 | 139.97 ± 42.87 | 64.60- 279.93 |
| Start frequency (Hz) | 782.84 ± 60.67 | 646.00- 861.33 | 608.08 ± 85.36 | 409.13- 750.00 | 617.91 ± 70.66 | 473.73- 732.13 | 491.42 ± 61.20 | 409.13- 667.53 |
| Middle frequency (Hz) | 707.12 ± 57.59 | 516.80- 861.33 | 682.96 ± 50.49 | 538.33- 818.26 | 636.63 ± 82.08 | 516.80- 775.20 | 554.48 ± 49.60 | 409.13- 646.00 |
| End Frequency (Hz) | 632.80 ± 56.55 | 516.80- 732.13 | 607.22 ± 71.59 | 495.26- 753.66 | 653.49 ± 67.04 | 516.80- 775.20 | 566.02 ± 65.22 | 430.66- 667.53 |
| Frequency change over time (Hz) | -150.04 ± 55.55 | -258.40- -43.07 | -0.86 ±  78.50 | -150.73- 193.80 | 35.58 ± 60.42 | -86.13- 129.20 | 74.60 ± 79.39 | -129.20- 236.86 |
| Total Slope (Hz/s) | -343.50 ± 147.90 | -836.57- -61.96 | 32.00 ±  205.53 | -279.35- 819.45 | 163.97 ± 338.17 | -669.25- 708.33 | 305.12 ± 314.83 | -296.87- 1028.70 |
| Slope 1^st^ half (Hz/s) | -355.28 ± 332.21 | -1673.10- 169.48 | 313.95 ± 442.02 | -558.70- 1609.40 | 92.60 ± 663.22 | -2006.20- 1305.00 | 437.79 ± 505.05 | -975.46- 1485.00 |
| Slope 2^nd^ half (Hz/s) | -331.71 ± 232.23 | -969.53- 0.00 | -249.92 ± 388.28 | -1179.60- 1251.90 | 235.64 ± 450.66 | -743.38- 944.44 | 172.44 ± 630.73 | -1058.10- 2192.30 |
| Pulse Rate (pulses/  minute) | 56.13 ± 4.65 | 45.00-  60.00 | 54.93 ± 4.69 | 40.00-  60.00 | 56.09 ± 4.99 | 50.00-  60.00 | 55.89 ± 4.09 | 50.00-  60.00 |

| **FORMANT DISPERSION** | | | | | | | | |
| --- | --- | --- | --- | --- | --- | --- | --- | --- |
| Maximum frequency of second dominant frequency band (Hz) | 1364.20 ± 62.76 | 1205.90-1464.3 | 1227.20 ± 133.71 | 947.46 ± 1546.90 | 1320.70 ± 100.39 | 1162.80- 1550.40 | 1057.40 ± 36.29 | 968.99- 1141.30 |
| Formant dispersion between bands (Hz) | 662.67 ± 79.28 | 430.66- 861.33 | 558.03 ± 117.54 | 279.93 ± 843.75 | 668.55 ± 81.28 | 516.80- 818.26 | 501.42 ± 54.93 | 409.13- 710.60 |

**Table B: Eigenvalues and variance of principal components for primary roars**

| **Principle component** | **Eigenvalue** | **Variance percent** | **Cumulative variance percent** |
| --- | --- | --- | --- |
| PC1 | 7.76 | 33.74 | 33.74 |
| PC2 | 3.59 | 15.62 | 49.36 |
| PC3 | 3.00 | 13.06 | 62.42 |
| PC4 | 1.94 | 8.42 | 70.84 |
| PC5 | 1.40 | 6.10 | 76.94 |

*
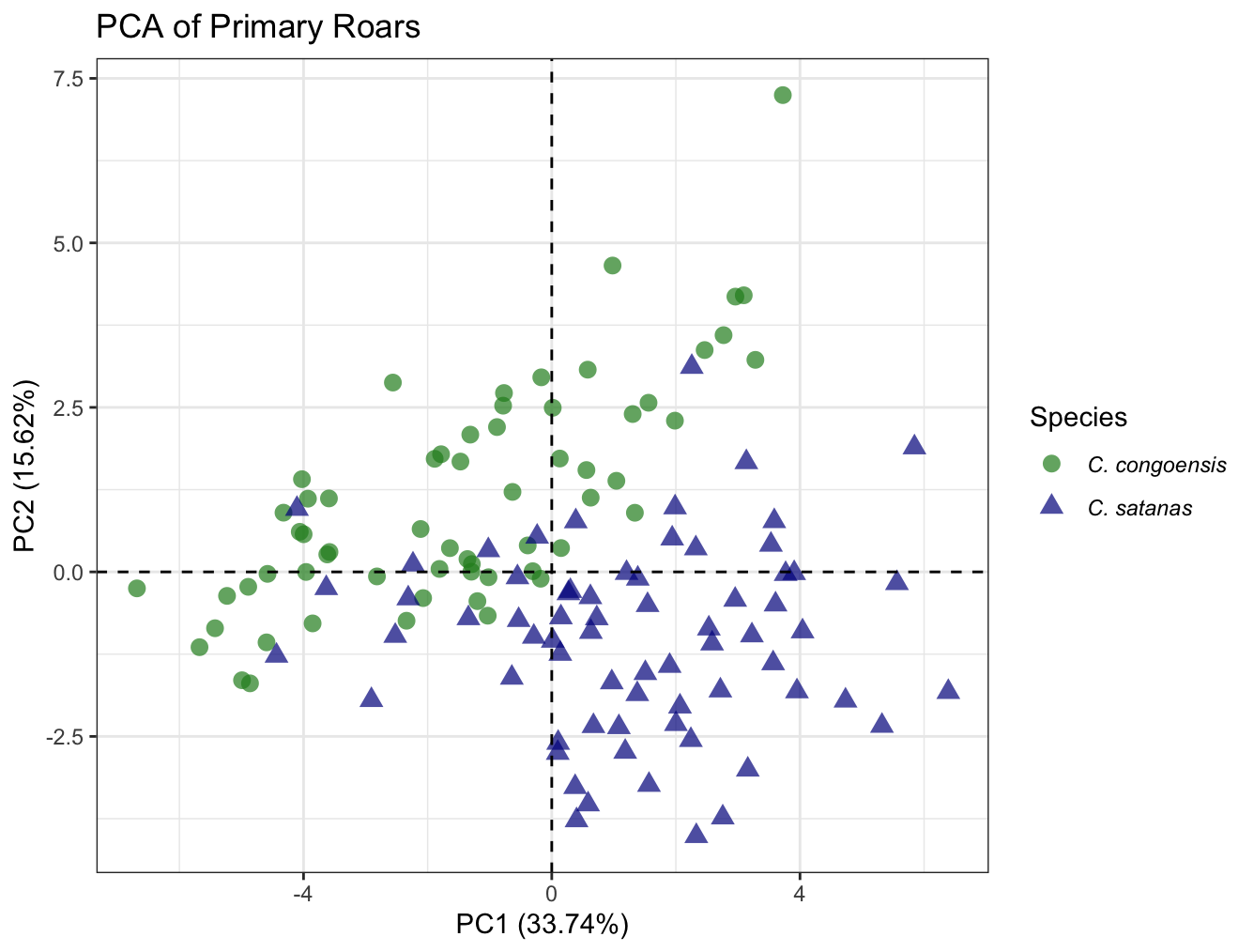
*

**Figure A. PCA of primary roars**

**Table C: Loadings of primary roar parameters on PC1 and PC2**

| **Parameter** | **PC1** | **PC2** |
| --- | --- | --- |
| **TOTAL CALL** | | |
| Maximum frequency | **-0.27** | -0.11 |
| 25% frequency | -0.26 | -0.22 |
| 50% frequency | **-0.30** | -0.20 |
| 75% frequency | -0.15 | -0.12 |
| Rise time | 0.08 | -0.09 |
| **FUNDAMENTAL FREQUENCY BAND** | | |
| Duration | 0.08 | -0.10 |
| Lower frequency limit | 0.16 | -0.08 |
| Upper frequency limit | -0.23 | 0.06 |
| 25% frequency | -0.25 | -0.22 |
| 50% frequency | **-0.32** | -0.16 |
| 75% frequency | **-0.30** | -0.11 |
| Maximum frequency | **-0.27** | -0.11 |
| Interquartile range | 0.06 | 0.17 |
| Start frequency | **-0.27** | **0.30** |
| Middle frequency | -0.20 | -0.21 |
| End Frequency | -0.23 | -0.03 |
| Frequency change over time | 0.15 | **-0.36** |
| Total Slope | 0.14 | **-0.37** |
| Slope 1^st^ half | 0.16 | **-0.43** |
| Slope 2^nd^ half | -0.03 | 0.10 |
| Pulse Rate | -0.09 | -0.07 |
| **FORMANT DISPERSION** | | |
| Maximum frequency of second dominant frequency band | -0.25 | 0.22 |
| Formant dispersion between bands | -0.15 | **0.29** |

Bolded values represent parameters that load highest for each component

**Table D: Eigenvalues and variance of principal components for secondary roars**

| **Principle component** | **Eigenvalue** | **Variance percent** | **Cumulative variance percent** |
| --- | --- | --- | --- |
| PC1 | 10.05 | 43.70 | 43.70 |
| PC2 | 3.17 | 13.79 | 57.48 |
| PC3 | 3.00 | 13.05 | 70.53 |
| PC4 | 1.86 | 8.07 | 78.60 |
| PC5 | 1.37 | 5.94 | 84.54 |


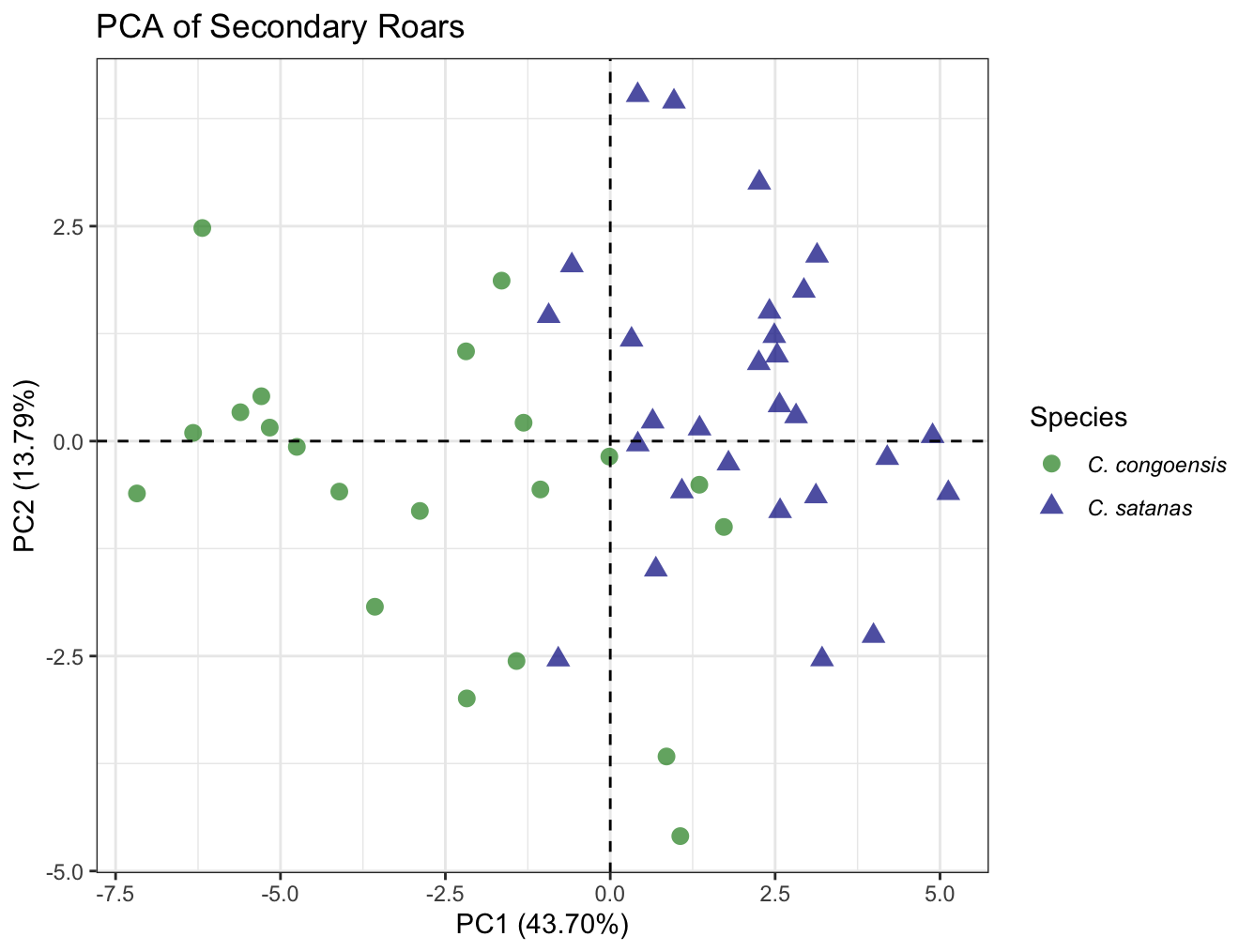


**Figure B. PCA of secondary roars**

**Table E: Loadings of secondary roar parameters on PC1 and PC2**

| **Parameter** | **PC1** | **PC2** |
| --- | --- | --- |
| **TOTAL CALL** | | |
| Maximum frequency | -0.23 | 0.09 |
| 25% frequency | **-0.30** | 0.04 |
| 50% frequency | **-0.29** | 0.07 |
| 75% frequency | -0.11 | 0.05 |
| Rise time | 0.03 | -0.09 |
| **FUNDAMENTAL FREQUENCY BAND** | | |
| Duration | 0.03 | -0.09 |
| Lower frequency limit | 0.04 | **0.22** |
| Upper frequency limit | -0.26 | 0.04 |
| 25% frequency | **-0.29** | 0.03 |
| 50% frequency | **-0.31** | 0.05 |
| 75% frequency | **-0.30** | 0.04 |
| Maximum frequency | **-0.30** | 0.06 |
| Interquartile range | 0.11 | -0.00 |
| Start frequency | -0.26 | **-0.23** |
| Middle frequency | **-0.29** | 0.04 |
| End Frequency | -0.24 | 0.20 |
| Frequency change over time | 0.05 | **0.51** |
| Total Slope | 0.04 | **0.53** |
| Slope 1^st^ half | -0.01 | **0.41** |
| Slope 2^nd^ half | 0.06 | 0.16 |
| Pulse Rate | -0.04 | 0.09 |
| **FORMANT DISPERSION** | | |
| Maximum frequency of second dominant frequency band | -0.27 | -0.13 |
| Formant dispersion between bands | -0.16 | **-0.23** |

Bolded values represent parameters that load highest for each component
