# Supplementary material for: Likweli: A remarkable new species of Colobus monkey from the Lomami National Park, Democratic Republic of Congo
Source: PLoS One. 2026 Jul 15;21(7):e0349857. doi: 10.1371/journal.pone.0349857 (PMC13372154; doi:10.1371/journal.pone.0349857)
Supplement: S3 File — (DOCX) [file pone.0349857.s003.docx]

# Extended description of field survey methods

**Text A:** Methods used to discover and document *Colobus congoensis.*

Field surveys had two objectives: First, detection of *C. congoensis* with associated opportunistic observations; Second, evaluation of *C. congoensis* relative abundance. We used five methods to survey *C. congoensis*, described below. Table A provides a comparative summary of the methods.

**Terrestrial Surveillance Patrols**

The Terrestrial Surveillance Patrols (TSP) were used throughout the park. Between 2020 and 2022, spatial and temporal coverage of the area intersecting the range of the *C. congoensis* was augmented to increase likelihood of C. *congoensis* detections (Figure 9).

TSP were conducted on foot for comprehensive monitoring of large mammals including primates; TSP trajectories were planned by GIS, using a 5 × 5 km grid. They were executed in the field along compass-guided reconnaissance tracks, termed recces, documented by GPS track logs. Deviations from the planned path were undertaken to follow up on selected observations, after which the planned trajectory was resumed. TSP were led by technicians trained to identify and document observations of fauna and to evaluate indicators of human activities. They were accompanied by ICCN park guards and local guides. Patrol teams progressed at a rate of 5 to 7 km per day. Patrol missions averaged 12 days and covered on average 75 km of reconnaissance track.

Primate groups were detected by audition (vocalization, vegetation movement) or by direct sighting. If the primate group detection was not a direct visual observation, two or three trained observers left the recce track to make visual contact with the group.  Group composition, position in the vegetation stratum and forest type (determined by canopy tree species), understory density and substrate class were recorded.  Observations of primate infants and other behaviors were recorded opportunistically.  Photos and videos were taken as possible to confirm species identities and document behaviors. Observers spent from 5 to 45 minutes (average 12 minutes) in visual contact with the group before returning to the line of travel and continuing on the planned trajectory. Geographic coordinates and habitat notes were taken for each observation at the point of detection.

Survey effort and spatial coverage by TSPs were evaluated using GIS. Tracklog distance for intersected grid cells was determined for each patrol.  Grids with high TSP coverage were defined as those having at least three visits by patrol team per year between 2020 and 2022, and with total summed patrol track of at least 30 km.

TSP used a consistent methodology to ensure comparable and comprehensive coverage of the LNP area. The method was constrained, however, by limited time spent for each detection, in particular searches off the line of travel.

**Line Transect Inventories**

Line transect Inventories (LTI) were conducted between 2020 and 2022 on six mapped blocks in *C. congoensis* range ranging in area from 53 to 129 km^2^. LTI blocks covered major habitat classes in the range area of *C. congoensis*, and included areas where *C. congoensis* had been detected by other methods as well as areas where there were no antecedent detections (Figure 10).

Transect layouts were determined by GIS and consisted of minimally cleared tracks along a pre-determined compass bearing; lines were separated by 1000 m. Field teams composed of a leader with compass and GPS and accompanied by two or three additional observers, slowly walked the transect line, starting in the early morning listening and looking for primates. GPS coordinates of all primate detections were recorded and classified as auditory only or auditory and visual. Teams temporarily left the transect line to collect data on group composition and habitat as described for TSP.

Most inventories for a given block covered multiple days. For the three largest inventory blocks, field work was conducted, by different teams to achieve coverages of the established transect lines. LTI effort was defined for each block as the summed surveyed transect length across all observer teams.

Following surveys, observations were mapped, and coded for species composition, and group sizes of component species.

**Dawn call surveys**

Dawn call surveys (DCS) were a method used throughout the park. Survey effort was defined as the summed number of individual surveys between 2020 and 2022 in each of mapped 5 × 5 km grid cells intersecting the range of *C. congoensis* (Figure A).

DCS were conducted to determine the relative frequency of detection of primate species which routinely emit loud call vocalizations at dawn. DCS were conducted during TSP, and LTI before field work was initiated for the day. During DCS three observers familiar with the vocalizations of primates in LNP, stationed themselves at a concealed location at least 200 meters from the bivouac camp. DCS were initiated between 5h30 and 6h15 and lasted 30 minutes.  Weather conditions were noted. Surveys were not done during falling rain or when rain threatened. All identified primate calls detected during the count period were confirmed to identity by recorders. Compass bearing of detected calls and number of calls during the calling bout were recorded. Calls were classified by proximity as immediate (primates both heard and seen, in which case species identities were confirmed visually, and number seen were recorded), proximal (call was clear and loud, but there was no visual detection), or distant (call had reduced amplitude and clarity, but was still identifiable). Detections for which the species identity was uncertain were not recorded.

**Riverine monitoring patrols**

Riverine monitoring patrol (RMP) survey effort was defined as the summed distance covered between 2019 and 2022 along the section of the Lomami River that intersected the range of *C. congoensis*.

Three to four observers in a motorized dugout moved slowly along the river and recorded vocalization or visual detection of animals in vegetation on the bank of the river. For each observation, GPS coordinates, the side of the river (east or west bank), species identity and, for visual detections, number seen were noted.

**Directed Searches**

Directed searches (DS) were used to confirm *C. congoensis* locations provided by local informants, to get photos of the species and to make recordings of vocalizations. During DS two or three observers, usually accompanied by a local guide with knowledge of *C. congoensis*, slowly moved through an area where the species was known or suspected to occur. Observers moved quietly watching and listening for primate groups. Locations of detections were recorded by GPS. Observers discreetly approached groups to determine species identities and numbers of individuals. Given the highly varied contexts of DS, no measure of survey effort was determined for the method.

**Table A: Summary of methods used to document and survey *Colobus congoensis*, 2018 – 2022**.

| **Method and period used** | **Description** | **Data collected** | **Measure of survey effort** | **Advantages** | **Constraints** | **Modifications for  *C. congoensis* surveys** |
| --- | --- | --- | --- | --- | --- | --- |
| Terrestrial surveillance patrol  (2018 - 2022) | Georeferenced observations from pre-planned reconnaissance tracks. | - Location primate detections. - Group size, group composition. | Spatial (track distance) and temporal (number annual patrols) coverage of 5 x 5 km grid intersecting known range. | - Method used over entire LNP since 2016. - Trained and experienced staff. - Multiple teams survey large area with consistent methodology. | - Limited time to document detections. - Few searches off planned track line. | - Observers leave recce track to make contact with detected primate groups. - Increased spatial and temporal coverage in *C. congoensis* range area. |
| Line transect inventory (2020 – 2021) | Georeferenced observation from systematically placed transect lines in defined blocks | - Location primate detections. - Group size, group composition. | Transect distance summed over replicate surveys for each block. | Estimates of encounter rates comparable across blocks. | Number of replicated surveys varied between blocks. | Observers leave transect to make contact with detected primate groups. |
| Dawn call counts (2020 – 2022) | Point counts of vocalizations made from fixed points during a defined period. | - Location primate detections. - Relative frequency detection. | Number of independent surveys. | - Standardized methods. - Comparison of detection frequency with other primates. | Possible confusion between *C. congoensis* and *C angolensis* vocalizations. | Use of observers able to distinguish *C. congoensis* calls. |
| Riverine monitoring patrol (2019 – 2022) | Riverside search for primates from motorized dugout on Lomami River. | - Location, group size - Presence of other primates | Distance covered along river course. | Access to locations and environments not accessible by ground-based observers. | - limited time to document detection. - Primates may flee before boat passes. | Boat maneuvered to permit observation. |
| Directed search (2018 – 2022) | Searches made with local guides in areas of suspected *C. congoensis* occurrence. | - Location, group size - Associated behavior | None. | - Confirm local knowledge of species - Used for photography, vocalization recordings | Limited spatial coverage | Specific sites where C*. congoensis* occurrence is reported are targeted. |


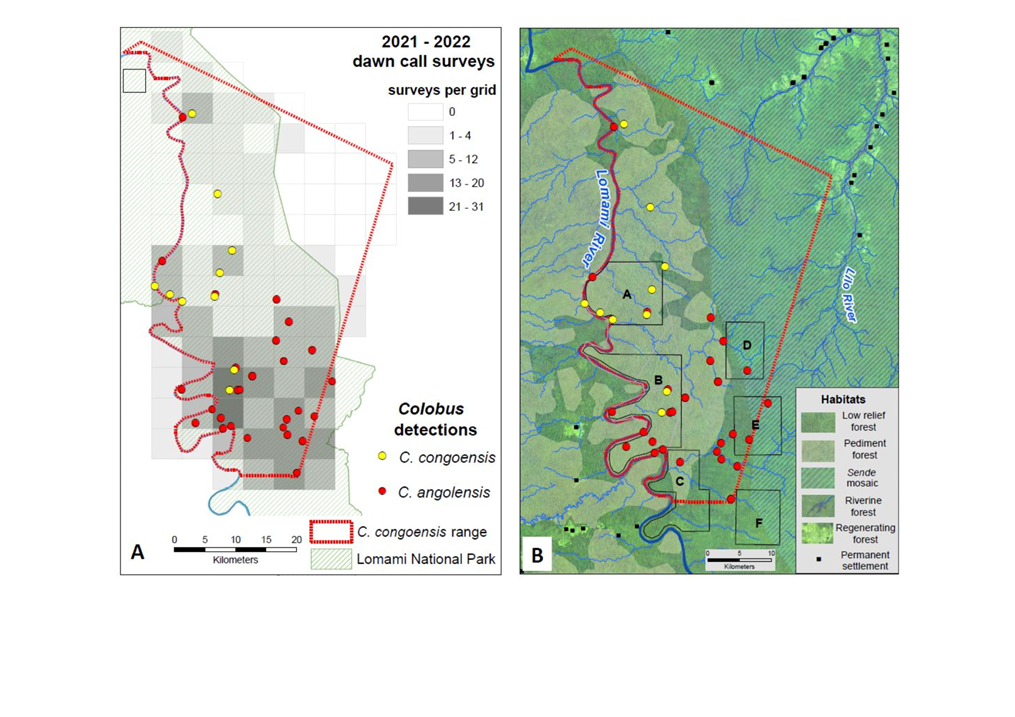


**Figure A.** Detections of *Colobus congoensis* and *C. angolensis* on dawn primate call surveys conducted between 2021 and 2022. **A)** Detections in relation to call survey coverage on 5 × 5 km grid intersecting *C. congoensis* range (n = 351 surveys). **B)** Distribution of detections in relation to habitat class. Habitat classes as in Figure 10.
