# Supplementary material for: Likweli: A remarkable new species of Colobus monkey from the Lomami National Park, Democratic Republic of Congo
Source: PLoS One. 2026 Jul 15;21(7):e0349857. doi: 10.1371/journal.pone.0349857 (PMC13372154; doi:10.1371/journal.pone.0349857)
Supplement: S4 File — (DOCX) [file pone.0349857.s004.docx]

# S4 file

## Text A: Extended descriptions of the skins and skulls of the *Colobus congoensis* specimens, with comparisons to *C. satanas*

Descriptions of the skins and skulls of individual *Colobus congoensis* specimens. There are 3 specimens of *C. congoensis*, 1 adult male, 1 old adult female with worn dentition, and 1 adult female with damage to the right lateral orbital margin and right side of face.

**YPM MAM 17307** (**JH30) Holotype:** Adult male, 6.9 kg, intermembral index (IMI) = 79.

Skin: Hair across head, tail, dorsum, and limbs is all jet black (Color 89 in Smithe, 1975) with no banding. Each hair is variably slightly lighter at the base, but otherwise jet black from base to tip over the entire skin. On top of the head, there are long hairs that stand straight up, similar to a crest or crown, ~5-6 cm long. The face mask around the eyes is lighter in color, grayish, light to pale neutral gray (Colors 85-86 in Smithe, 1975), with pale or peach colored skin around the mouth and upper lip. The hair on the dorsum is relatively long but noticeably shorter compared to that of *C. satanas*, ~9-12.5 cm along the mid-dorsum (see Figure D). At the base of the tail, there is no obvious tail tuft or extension of long dorsum hair onto the proximal portion of the tail (unlike in *C. satanas*). At the base of the tail, hair is ~2-3 cm long, and the middle of the tail hair is ~1 cm long. There is no observable tuft at the end of the tail in the study skin, although a small terminal tail tuft was noted during life (S2 Table).

Skull: Young adult male with relatively unworn dentition (Figure A). Upper and lower third molars are erupted; upper and lower incisors and canines are present but isolated from the alveoli. Upper canines appear to have not been quite fully erupted when they are placed back in the alveoli; they are very close to being fully erupted, but there is some space between the alveolus and the tooth. Lower canines appear fully erupted when placed back in the alveoli.

The left superior orbital margin is asymmetrical, and the torus is receded to expose more of the orbit superiorly, suggesting a healed injury.  The temporal lines are symmetrical along the neurocranium, but posteriorly the left nuchal crest is less developed than the right.  Additional postmortem damage to the left medial orbital margin extending into the nasal aperture is present.  The rostrum is short and square in superior view, with distinct canine jugae present in the maxillae. No maxillary or suborbital fossae are present. The malar region is shallow, and the maxillary alveolar process is short. Three infraorbital foramina are present on each side. There is slight bulging or bossing of the frontal above the orbits in YPM MAM 17307, reminiscent of the “saddle-shaped” frontal in *C. satanas*, although not as exaggerated. The temporal lines are moderately rugose anteriorly and run parallel along the neurocranium, remaining widely spaced until they converge very slightly towards inion. The nuchal crest is moderately developed, forming a low but distinct crest along the posterior margin of the skull. The nuchal crest undulates slightly superiorly in the midline. The nuchal plane is almost vertically oriented when the cranium is held in Frankfurt horizontal. The lateral pterygoid plates lack a posterior foramen, a feature most often observed in *Piliocolobus* rather than *Colobus*. The vomer does not extend far posteriorly, and the alae are abbreviated, forming a broad “U”-shaped posterior margin. The glenoid fossae are broad and shallow, and the postglenoid processes are small to non-existent. The external auditory meatus is short, ending well medial to porion.

The mandible is relatively shallow, and the symphysis is moderately sloping but likewise shallow. Lingually, the superior and inferior mandibular tori are weakly developed, and two lingual mental foramina are visible, placed horizontally. No median mental foramen is present anteriorly. The corpus is relatively even in depth moving posteriorly without *prominentia laterales* buttressing the corpus. Gonion is expanded. The ramus is short and relatively broad; the coronoid process is slightly taller than the condyle. Distinct mesial buccal clefts are present on the molars. The M_3_s have large, slightly buccally positioned hypoconulids.

**YPM MAM 17306 (JH28) Paratype:** Adult female, 7.4 kg, IMI = 75.

Skin: Hair across head, tail, dorsum, and limbs is all jet black (Color 89 in Smithe, 1975). Individual hairs are again entirely black, slightly lighter at their base, with no banding. On top of the head, there are long hairs that stand straight up, similar to a crest or crown, ~4-5 cm long. Face mask around the eyes is lighter in color, grayish, light to pale neutral gray (Colors 85-86 in Smithe, 1975), with pale or peach colored skin around the mouth and upper lip (as assessed from field photos). The hair on the dorsum is relatively long for its body size but noticeably shorter compared to that of *C. satanas*, ~5-7.5 cm along the mid-dorsum. At the base of the tail, there is no obvious tail tuft or extension of the long dorsum hair to the proximal portion of the tail (unlike in *C. satanas*). At the base of the tail, hair is ~2-3 cm long, and the middle of the tail hair is ~1 cm long. There is no tuft at the end of the tail.

Skull: Adult female with moderate to heavy dental wear (Figure B). Dentition is intermediate in wear, not as worn as in female YPM MAM 17308 but more worn than in male YPM MAM 17307. This individual has healed damage to the right orbit on the lateral margin and right side of face, suggesting a severe injury in life with significant bone resorption. The palate lacks P^3^s, although no injury and/or alveolar resorption is evident, so this morphology seems independent of the injury. The mandibular dentition is complete, and this asymmetry has produced a significant underbite with substantial subnasal prognathism such that the incisors project almost anteriorly. The canine is worn on the distal surface and likely apically too but is still very small, barely projecting below the occlusal plane of the postcanine teeth (Figure B). The temporal lines are again rugose and widely spaced anteriorly but converge just above inion. There is no sagittal crest, and the nuchal crest is modest but extends nearly continuously across the posterior margin of the neurocranium. Because the specimen is damaged, it is unclear if this nuchal morphology is typical or not. On the basicranium, the lateral pterygoid plates form a foramen along the posterior margin. The postglenoid processes are average in height, most similar to those of YPM MAM 17308, and the auditory meatus is short, reaching laterally to approximately the level of the postglenoid process.

In the mandible, the corpus deepens only slightly posteriorly, and the gonion is slightly expanded. No *prominentia laterales* are present. The ramus is moderately tall with a shallow coronoid notch and a tall coronoid process. A superior transverse torus extends along the lingual surface of the corpus, and an inferior transverse torus is present in the midline of the symphysis. The dentition exhibits very large, broad mesiobuccal clefts on the premolars and molars. The M_3_ is unreduced, but the hypoconulid is relatively small.

**YPM MAM 17308 (JH31) Paratype:** Adult female, 7.4 kg, IMI = 76.

Skin: Hair across head, dorsum, and limbs is all jet black (Color 89 in Smithe, 1975). The tail has some gray (Glaucous 79-80 in Smithe, 1975) hairs in the dried skin (which could be a slightly different shade of gray in life) starting ~22-23 cm from the base of the tail (i.e., the proximal quarter of the tail is jet black, but the distal 3/4 of the tail exhibits grayish hairs). Field photos illustrate grayish hair on the lower back and tail in some individuals, which could be due to older age. Other than the gray hairs described above, individual hairs are typically entirely black from base to tip, slightly lighter in color at the base, with no banding. On the top of the head, there are again long hairs that stand straight up, similar to a crest or crown, ~4-5 cm long. The face mask around the eyes is lighter in color, grayish, light to pale neutral gray (Colors 85-86 in Smithe, 1975), with pale or peach colored skin around the mouth and upper lip (as assessed from field photos). The hair on the dorsum is relatively long for its body size but noticeably shorter in absolute terms compared to that of *C. satanas*, ~6-8 cm along the mid-dorsum. At the base of the tail, there is no obvious tail tuft or extension of the long dorsum hairs to the proximal portion of the tail (unlike in *C. satanas*). Base of the tail hair is ~2-2.5 cm long, and the middle of the tail hair is ~1-1.5 cm long. There is no tuft at the end of the tail.

Skull: Adult female with heavily worn dentition (Figure C). The canines are small, even for a female cercopithecid monkey. The M_3_s are slightly reduced distally, perhaps lacking a full hypocone, but as the dentition is very worn this is hard to ascertain beyond crown outline. The presence of mesial buccal clefts on the lower molars is also unobservable due to the heavy wear. The lower left P_4_ is abnormally worn nearly to the roots from the buccal side, but molar wear does not seem excessively asymmetrical. The rostrum is very abbreviated with moderate subnasal prognathism. No maxillary fossae are present, though there is slight development of an infraorbital ridge. Three infraorbital foramina are present on the left side and two are present on the right side. The temporal lines originate from the lateral margin of the orbit and are well developed, converging very slightly to a more medial position as they remain widely separated while moving posteriorly to the back of the cranium. A slight nuchal crest is present at the back of the skull, restricted to the lateral third of the neurocranium, and forms a straight line with no undulations. Inferiorly, the postglenoid processes are mediolaterally narrow but moderately tall, closely appressed to the external auditory meatus, which extends more laterally to nearly meet porion. The vomer is posteriorly abbreviated, lacking extension of the alae and forming a straight posterior margin.

The mandibular corpus deepens slightly posteriorly, but there is no notable gonial expansion in this specimen. There is slight development of the *prominentia laterales*, but not as prominent as in *Piliocolobus* and largely restricted to the posterior portion of the corpus. Lingually, the superior mandibular torus is moderately developed; the lingual mental foramina are obscured with tissue. There is no median mental foramen on the anterior surface of the symphysis. The ramus is relatively short with a slightly taller coronoid process compared to the condyle.

Description of skins of *C. satanas*. Six skins of *C. satanas* were examined, including two adult males (AMNH M-236358 and AMNH M-89381). The other skins are of unknown sex and possibly include two juvenile specimens although sex or age is not recorded. They lack associated skulls, which could provide additional age/sex information. All skins make it clear that the legs are longer than the arms. As described by Fleury and Brugiere (2013), the entire pelage and skin is black in the skins examined here. The hairs are jet black (Color 89 in Smithe, 1975), variably and occasionally ranging down to a black/neutral gray (color 82 in Smithe, 1975) to jet black at their base, although most similar to Jet Black (i.e., it is darker than color 82 in the specimens examined here). Intermembral index (IMI) in this species is ~79 (Rowe and Myers, 2013).

**AMNH M-236358** is from Cameroon, ~15 km W Eseka (see O’Leary, 2003 for details). Hairs on the dorsum are relatively long, ~12-16.5 cm from base to tip, in the middle of the dorsum. There is no banding on the hairs visible; the hair is black from the base to the tip over the entire skin, including the legs, tail, arms, and head. There is a crest of upright hairs on the top of the head that are arranged haphazardly. The long hairs on the dorsum run right up to the base of the tail and then continue along the most proximal portion of the tail, about 15-16 cm distally from the base, after which the hair is noticeably shorter (~1.5-3 cm). This gives the appearance of a “tuft” at the base of the tail (Fleury and Brugière, 2013). Moving distally, the short hairs on the tail, though still jet black, continue for the rest of the length of the tail. There is no tail tuft at the end of the tail.

**AMNH M-89381** is from Cameroon, Mfume area. Again, the hair is entirely black, closest to Jet Black (Color 89 in Smithe, 1975) from base to tip. This skin has some bare spots, particularly on the left arm and tail, most likely due to long storage. The hair on the dorsum is relatively long, ranging from ~12.5-16.5 cm on average from the base to the tip. A crest of upright hairs on the top of the head is present, and the hair at the base of the tail (or just above) is noticeably longer than the hair on the rest of the tail beginning ~15-18 cm from the base. From this point, the hair along the tail is shorter (~1.5-3 cm) in length from tip to the base. There is no distal tail tuft. This specimen appears a bit smaller than AMNH M-236358, but both are recorded as adult males.

**AMNH M-167652** is from Cameroon, South Efulan, 53 miles east of Kribi. It is similar in size to adult males above. The head, hands, and feet are preserved. The hair on top of the head appears matted and a bit shorter than in the two confirmed male specimens above, upright in parts but does not form as obvious a crest as in AMNH M-236358 and AMNH M-89381. Hair is black/neutral gray to jet black from base to tip. The hairs are relatively long on the dorsum but noticeably a little bit shorter than in the adult males. Measured in the middle of the dorsum, hairs are ~7-12.5 cm long from base to tip. The longer hair extends to just above the base of the tail and then gets shorter ~13 cm distal to the base of the tail, ~1.5-3.5 cm long from that point onwards. There is no distal tail tuft. Sex is not recorded for this specimen but it may be an adult female that has relatively shorter hair and perhaps not as much of a crest due to slight dimorphism in the pelage.

**AMNH M-167653** is from Cameroon, South Efulan, 53 miles east of Kribi. Sex is not recorded, but it is similar in size to the confirmed adult males. The head, hands, and feet have been removed and are not observable, precluding assessment of any sexual dimorphism in crown development. Hair is black/neutral gray to jet black from base to tip. The hairs are relatively long on the dorsum, ranging from 11.5-16 cm long from base to tip. The longer hair extends to above the base of the tail and then gets shorter ~15-18 cm distal to the base of the tail, ~1.5-3 cm long from that point onwards. There is no distal tail tuft.

**AMNH M-119763** is from Gabon, Kango Estuaire. This skin is noticeably smaller than the others, probably indicating that it is a juvenile or, less likely, a female (no sex or age is recorded). It is interesting, however, that both skins from Gabon are noticeably smaller, possibly because they are juveniles/females or possibly because this population is consistently smaller. Assuming it is a juvenile, it is again notable for having the same pelage as the adults: all black/neutral gray to jet black from base to tip. The head, hands, and feet have been removed, so they are not observable. The hairs are again relatively long on the dorsum, ~9.5-16 cm long from base to tip. The longer hair extends to above the base of the tail and then gets shorter ~19 cm distal to the base of the tail, ~1.5-3 cm long from that point onwards. There is no distal tail tuft.

**AMNH M-119764** is from Gabon, Kango Estuaire. The skin is noticeably smaller than the others, probably indicating that it is a juvenile or, less likely, a female (no sex or age is recorded). Assuming it is a juvenile, it is again notable for having the same pelage as the adults: all black/neutral gray to jet black from base to tip. The head, hands, and feet have been removed, so they are not observable. The hairs are again relatively long on the dorsum, ~9.5-14 cm long from base to tip. The longer hair extends to above the base of the tail and then gets shorter ~15 cm distal to the base of the tail, ~1.5-3 cm long from that point onwards. There is no distal tail tuft.

## References

Fleury, M.-C., Brugière, D., 2013. *Colobus satanas*. In: Butynski, T.M., Kingdon, J., Kalina, J. (Eds.), Mammals of Africa. Volume II: Primates. Bloomsbury Publishing, London, pp. 97–100.

O’Leary, R., 2003. An Annotated Catalog of the African Primate Genera *Colobus* and *Procolobus* (Cercopithecidae: Colobinae) in the Collections of the American Museum of Natural History. American Museum Novitates. 26.

Rowe, N., Myers, M., 2013. All the World’s Primates. Primate Conservation Inc., Charlestown.

Smithe, F., 1975. Naturalist’s Color Guide, Part 1. American Museum of Natural History, New York.


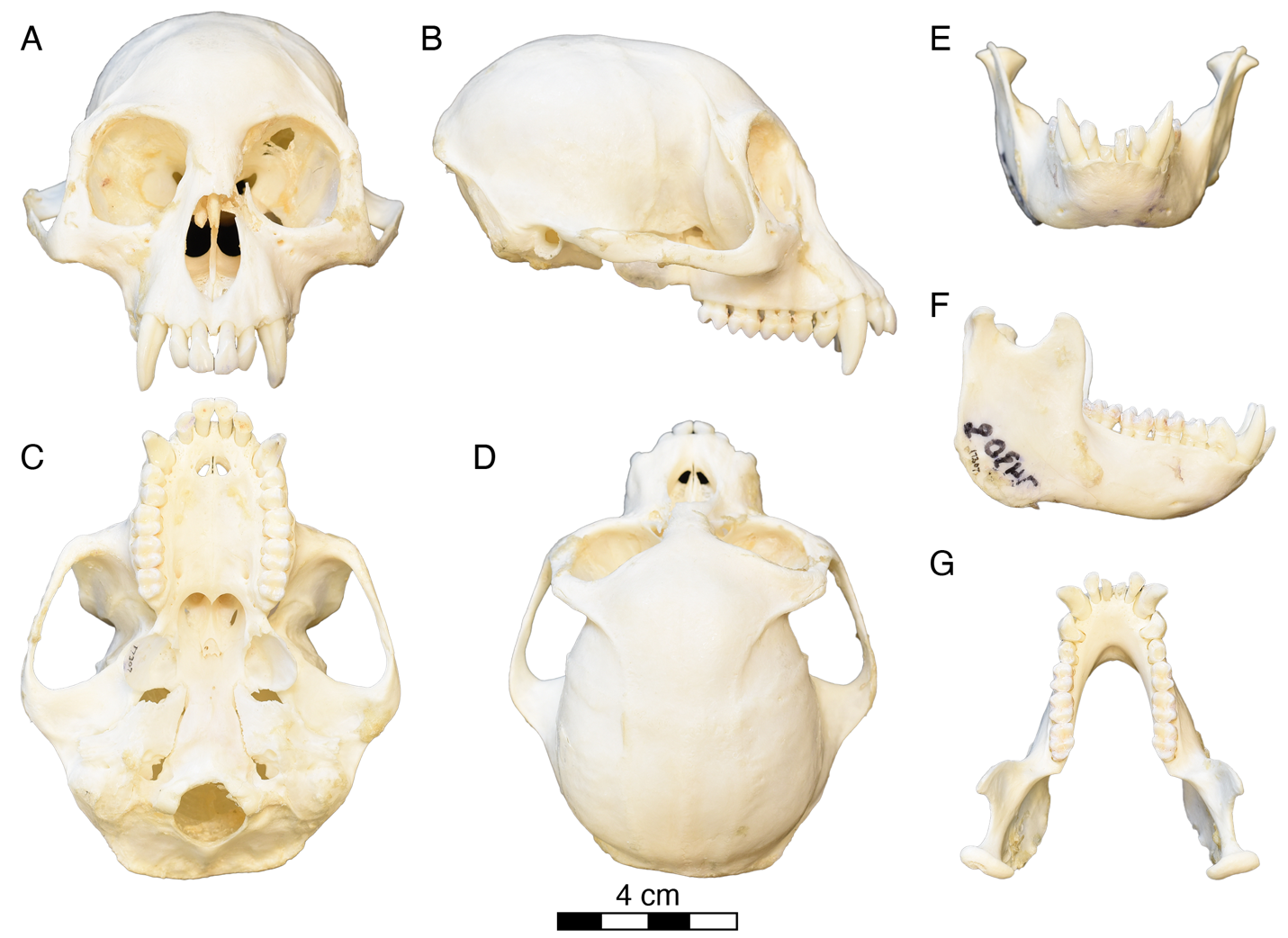


**Figure A.** Skull of *Colobus congoensis* holotype YPM MAM 17307 (male). Cranium in (A) anterior, (B) lateral, (C) occlusal, and (D) dorsal views, and mandible in anterior (E), lateral (F), and occlusal (G) views.


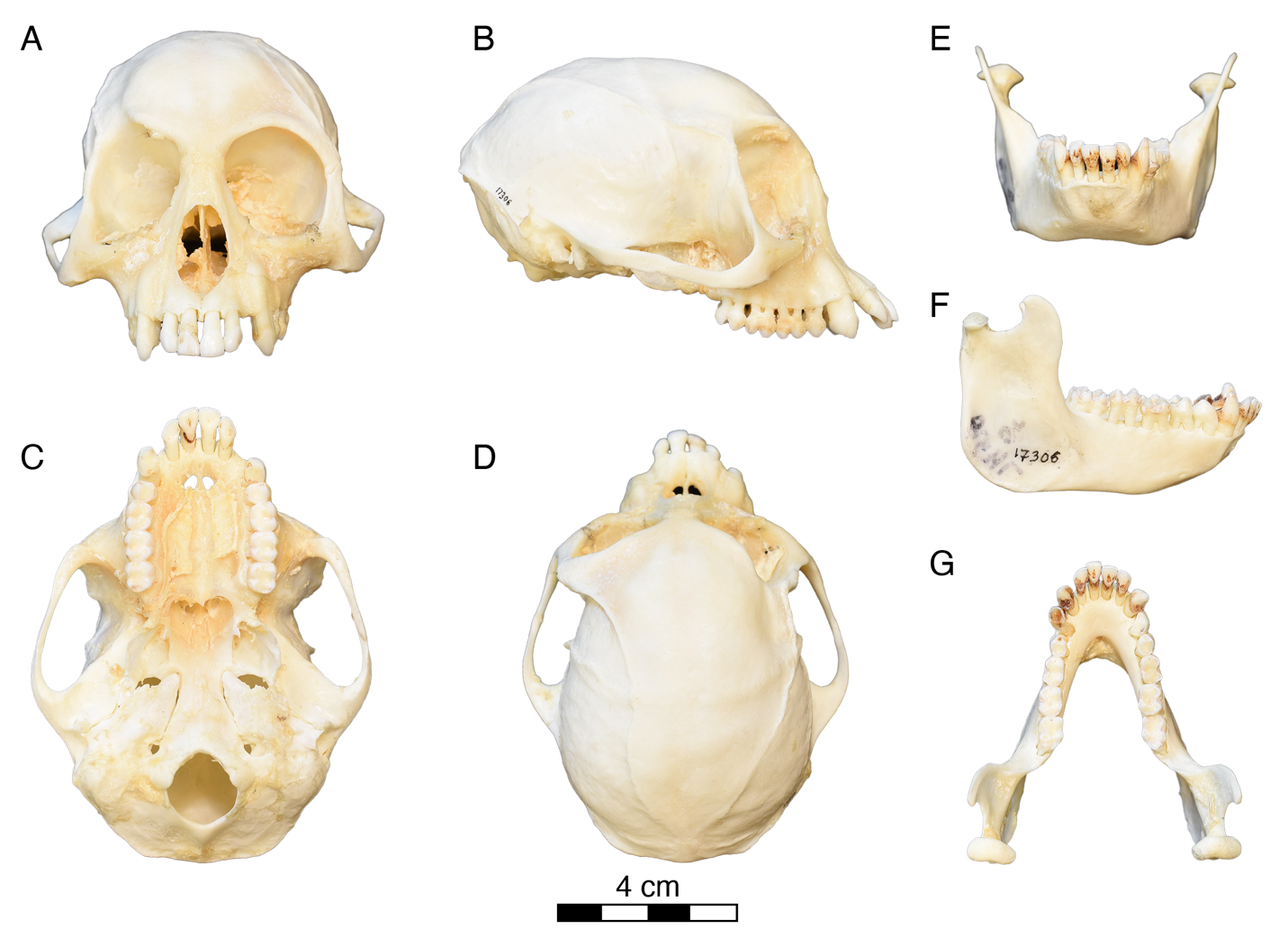


**Figure B.** Skull of *Colobus congoensis* paratype YPM MAM 17306 (female). Cranium in (A) anterior, (B) lateral, (C) occlusal, and (D) dorsal views, and mandible in anterior (E), lateral (F), and occlusal (G) views.


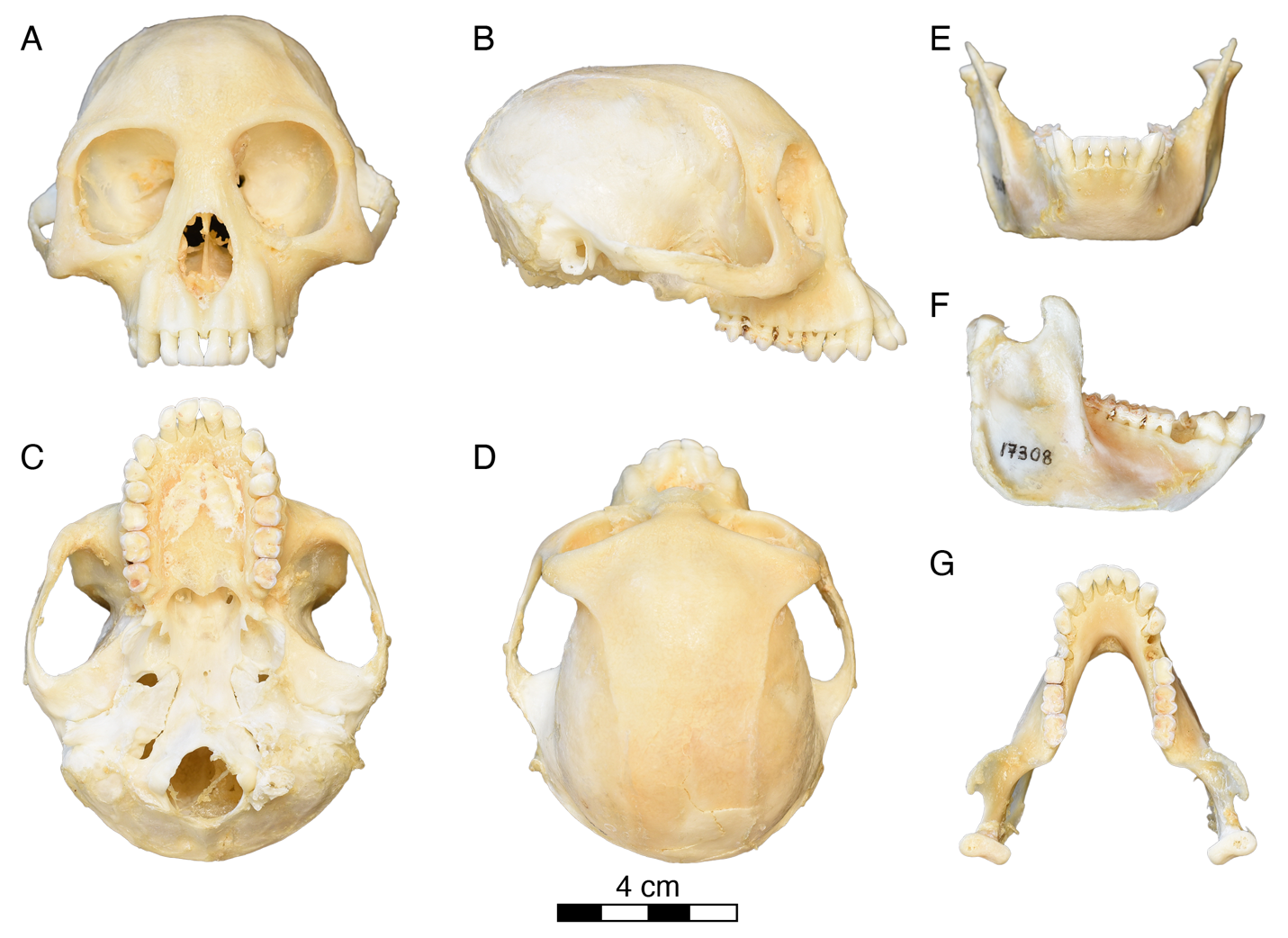


**Figure C.** Skull of *Colobus congoensis* paratype YPM MAM 17308 (female). Cranium in (A) anterior, (B) lateral, (C) occlusal, and (D) dorsal views, and mandible in anterior (E), lateral (F), and occlusal (G) views.

**
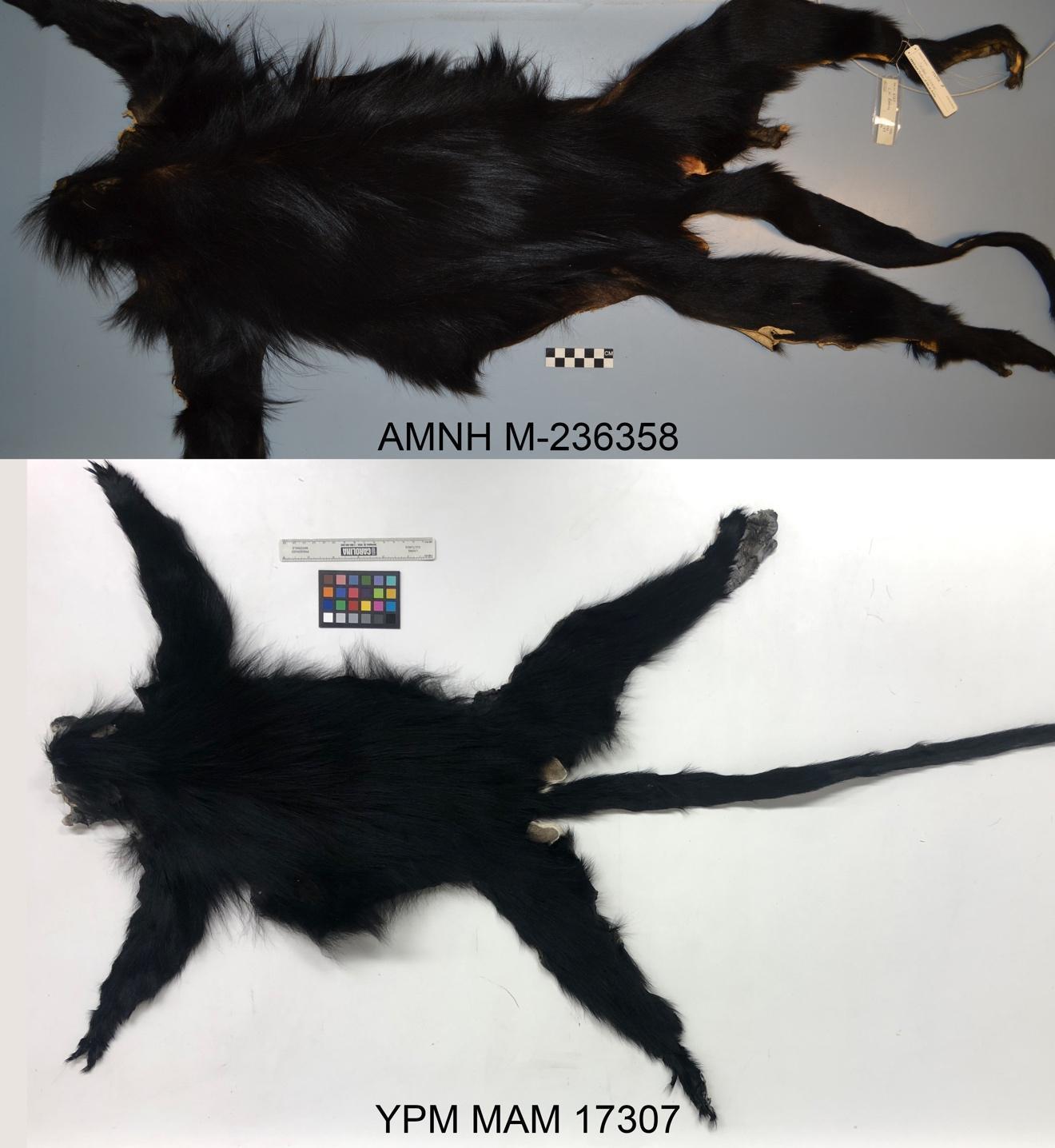
**

**Figure D.** Skin comparison between *Colobus satanas* and *C. congoensis* sp. nov. Skin of *C. satanas* adult male, AMNH M-236358 (top), compared with *C. congoensis* adult male holotype YPM MAM 17307 (bottom). Note the larger size of *C. satanas*, the longer hairs along the dorsum, and the longer hair forming a “tuft” at the base and most proximal portion of the tail. By contrast, *C. congoensis* is smaller, has shorter hairs on the dorsum, and lacks a tuft at the base of the tail. Skins are presented at the same scale.

**Table A:** Significance values of all post-hoc pairwise comparisons (Tukey’s Honestly Significant Differences) from ANOVAs of skeletal indices. Results and discussion of ANOVAs presented in the main text. Index definitions in Table 2 of main text.

**Petrous Shape**

|  | *C. congoensis* | *Colobus* | *Piliocolobus* | *Procolobus* |
| --- | --- | --- | --- | --- |
| *C. congoensis* |  |  |  |  |
| *Colobus* | 1 |  |  |  |
| *Piliocolobus* | 0.076 | 0 |  |  |
| *Procolobus* | 0.89 | 0.41 | 0.0020 |  |

**Palate Shape**

|  | *C. congoensis* | *C. satanas* | *C. angolensis* | *C. guereza* | *C. polykomos* | *C. vellerosus* |
| --- | --- | --- | --- | --- | --- | --- |
| *C. congoensis* |  |  |  |  |  |  |
| *C. satanas* | 0.59 |  |  |  |  |  |
| *C. angolensis* | 0.018 | 0.025 |  |  |  |  |
| *C. guereza* | 0 | 0 | 0 |  |  |  |
| *C. polykomos* | 0.059 | 0.20 | 0.95 | 0 |  |  |
| *C. vellerosus* | 0.00045 | 0.0012 | 0.12 | 0.91 | 0.043 |  |

**Corpus Depth**

|  | *C. congoensis* | *C. satanas* | *C. angolensis* | *C. guereza* | *C. polykomos* | *C. vellerosus* |
| --- | --- | --- | --- | --- | --- | --- |
| *C. congoensis* |  |  |  |  |  |  |
| *C. satanas* | 0.96 |  |  |  |  |  |
| *C. angolensis* | 0.12 | 0.060 |  |  |  |  |
| *C. guereza* | 0.0032 | 0.000045 | 0.099 |  |  |  |
| *C. polykomos* | 0.021 | 0.0025 | 0.74 | 0.87 |  |  |
| *C. vellerosus* | 0.11 | 0.15 | 0.95 | 1 | 1 |  |

**C^1^/M^1^ Area (males only)**

|  | *C. congoensis* | *C. satanas* | *C. angolensis* | *C. guereza* | *C. polykomos* | *C. vellerosus* |
| --- | --- | --- | --- | --- | --- | --- |
| *C. congoensis* |  |  |  |  |  |  |
| *C. satanas* | 1 |  |  |  |  |  |
| *C. angolensis* | 0.65 | 0.046 |  |  |  |  |
| *C. guereza* | 0.84 | 0.21 | 0.96 |  |  |  |
| *C. polykomos* | 0.64 | 0.046 | 1 | 0.94 |  |  |
| *C. vellerosus* | 0.86 | 0.74 | 1 | 1 | 1 |  |

**Cranial Size (pooled sex)**

|  | *C. congoensis* | *C. satanas* | *C. angolensis* | *C. guereza* | *C. polykomos* | *C. vellerosus* |
| --- | --- | --- | --- | --- | --- | --- |
| *C. congoensis* |  |  |  |  |  |  |
| *C. satanas* | 0.022 |  |  |  |  |  |
| *C. angolensis* | 0.00082 | 0.57 |  |  |  |  |
| *C. guereza* | 0.000078 | 0.069 | 0.80 |  |  |  |
| *C. polykomos* | 0.0011 | 0.64 | 1 | 0.79 |  |  |
| *C. vellerosus* | 0.000069 | 0.033 | 0.22 | 0.57 | 0.21 |  |

**I^1^/M^1^ Length**

|  | *C. congoensis* | *C. satanas* | *C. angolensis* | *C. guereza* | *C. polykomos* | *C. vellerosus* |
| --- | --- | --- | --- | --- | --- | --- |
| *C. congoensis* |  |  |  |  |  |  |
| *C. satanas* | 0.56 |  |  |  |  |  |
| *C. angolensis* | 1 | 0.0082 |  |  |  |  |
| *C. guereza* | 1 | 0.0023 | 1 |  |  |  |
| *C. polykomos* | 0.76 | 0.98 | 0.046 | 0.014 |  |  |
| *C. vellerosus* | 0.81 | 1 | 0.51 | 0.37 | 1 |  |

**M_2_ Shape (by genus)**

|  | *C. congoensis* | *Colobus* | *Piliocolobus* | *Procolobus* |
| --- | --- | --- | --- | --- |
| *C. congoensis* |  |  |  |  |
| *Colobus* | 0.021 |  |  |  |
| *Piliocolobus* | 0.99 | 0 |  |  |
| *Procolobus* | 0.26 | 0.021 | 0.00020 |  |

**M_2_ Shape (by species)**

|  | *C. congoensis* | *C. satanas* | *C. angolensis* | *C. guereza* | *C. polykomos* | *C. vellerosus* |
| --- | --- | --- | --- | --- | --- | --- |
| *C. congoensis* |  |  |  |  |  |  |
| *C. satanas* | 0 |  |  |  |  |  |
| *C. angolensis* | 0.13 | 0 |  |  |  |  |
| *C. guereza* | 0.13 | 0 | 1 |  |  |  |
| *C. polykomos* | 0.0051 | 0.000065 | 0.065 | 0.032 |  |  |
| *C. vellerosus* | 0.0062 | 0.092 | 0.18 | 0.15 | 0.99 |  |

**P^4^/M^1^ Length**

|  | *C. congoensis* | *Colobus* | *Piliocolobus* | *Procolobus* |
| --- | --- | --- | --- | --- |
| *C. congoensis* |  |  |  |  |
| *Colobus* | 0.0047 |  |  |  |
| *Piliocolobus* | 0.16 | 0 |  |  |
| *Procolobus* | 0.42 | 0 | 0.49 |  |

**Table B:** Sample sizes by subspecies and sex for six *Colobus* species with no missing data that could be included in multivariate analyses.

| **Subspecies** | **Females** | **Males** |
| --- | --- | --- |
| *Colobus angolensis angolensis* | 0 | 1 |
| *Colobus angolensis cottoni* | 6 | 6 |
| *Colobus congoensis* | 1 | 1 |
| *Colobus guereza dodingae* | 1 | 0 |
| *Colobus guereza kikuyuensis* | 6 | 3 |
| *Colobus guereza occidentalis* | 1 | 4 |
| *Colobus polykomos polykomos* | 5 | 8 |
| *Colobus satanas anthracinus* | 1 | 3 |
| *Colobus vellerosus* | 1 | 0 |

**Table C:** Pairwise Euclidean distances between pooled-sex means of eight of the nine craniomandibular indices presented in the text (excluding relative canine area; see Table 2 for description of indices), for six species of *Colobus* (including *C. congoensis*). *Colobus congoensis* is phenetically closest to *C. satanas*, but all other species of *Colobus* are phenetically closer to each other than any is to *C. congoensis*.

|  | ***C. congoensis*** | *C. satanas* | *C. angolensis* | *C. guereza* | *C. polykomos* |
| --- | --- | --- | --- | --- | --- |
| *C. satanas* | 2.9116121 |  |  |  |  |
| *C. angolensis* | 3.51953 | 0.6659668 |  |  |  |
| *C. guereza* | 4.2076621 | 1.3441804 | 0.7025836 |  |  |
| *C. polykomos* | 4.2947973 | 1.4043373 | 0.7798912 | 0.2299734 |  |
| *C. vellerosus* | 4.7221547 | 1.8299445 | 1.207944 | 0.5620138 | 0.4393088 |


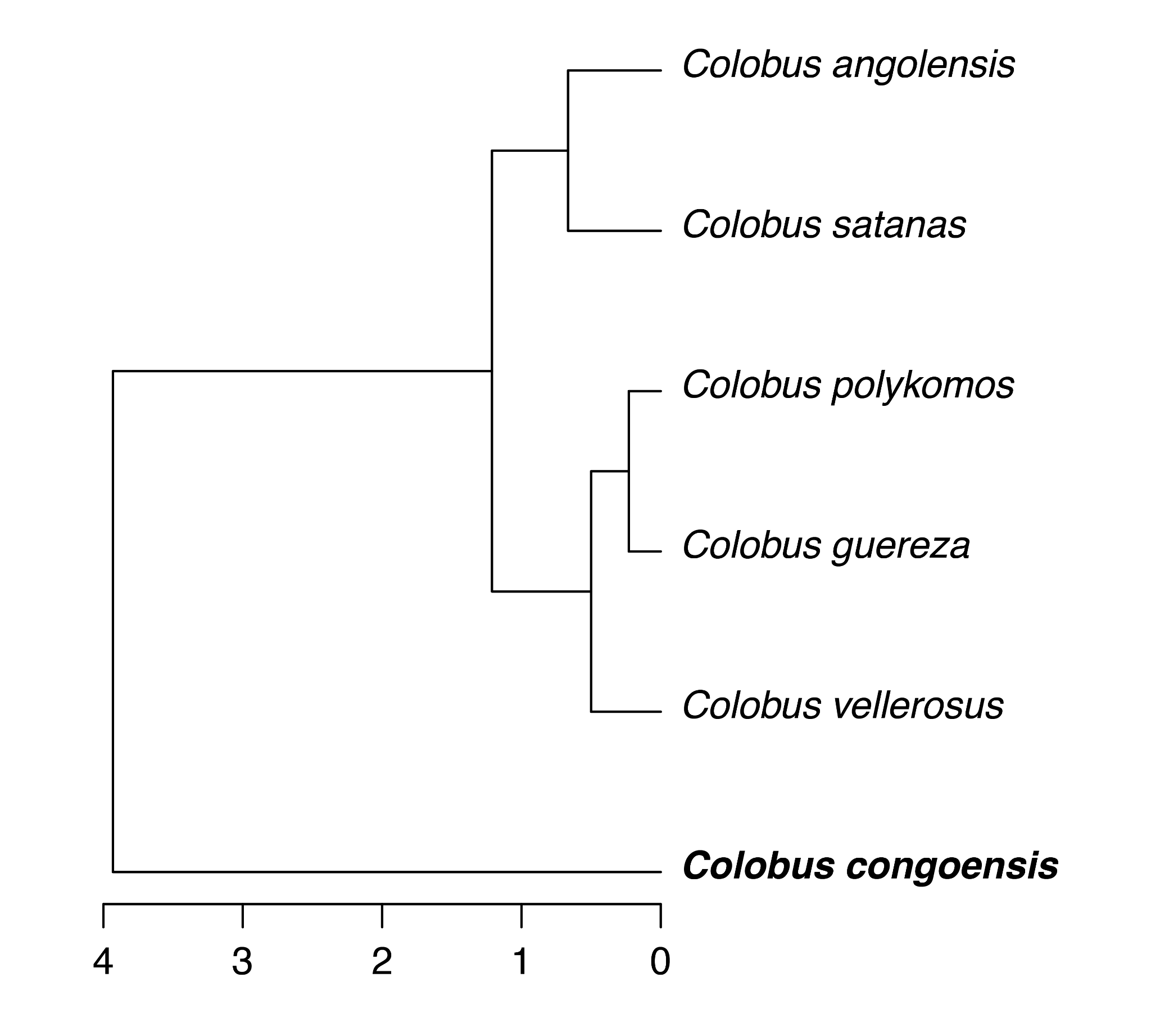


**Figure E**. UPGMA clustering dendrogram using the pairwise Euclidean distance matrix of *Colobus* species calculated from a pooled-sex sample of 8 craniomandibular indices (Table C). Dendrogram generated in R with *hclust()*. Scale reflects Euclidean distance. Cophenetic correlation coefficient = 0.9567.

**Table D:** Pairwise Euclidean distances between pooled-sex means of eight of the nine craniomandibular indices presented in the text (excluding relative canine area; see Table 2 for description of indices), for nine subspecies of *Colobus*. *C. congoensis* is again phenetically closest to *C. satanas* (for which only one subspecies is sampled).

|  | ***C. congoensis*** | *C. a. angolensis* | *C. a. cottoni* | *C. g. dodingae* | *C. g. kikuyuensis* | *C. g. occidentalis* | *C. p. polykomos* | *C. s. anthracinus* |
| --- | --- | --- | --- | --- | --- | --- | --- | --- |
| *C. a. angolensis* | 5.0045 |  |  |  |  |  |  |  |
| *C. a. cottoni* | 3.3965 | 1.6289 |  |  |  |  |  |  |
| *C. g. dodingae* | 4.4817 | 0.5759 | 1.0941 |  |  |  |  |  |
| *C. g. kikuyuensis* | 4.1205 | 0.9446 | 0.7376 | 0.3932 |  |  |  |  |
| *C. g. occidentalis* | 4.3118 | 0.8050 | 0.9303 | 0.2784 | 0.2326 |  |  |  |
| *C. p. polykomos* | 4.2948 | 0.7337 | 0.9045 | 0.2312 | 0.2752 | 0.2604 |  |  |
| *C. s. anthracinus* | 2.9116 | 2.1118 | 0.5594 | 1.5963 | 1.2633 | 1.4472 | 1.4043 |  |
| *C. vellerosus* | 4.7222 | 0.3708 | 1.3317 | 0.3024 | 0.6416 | 0.4946 | 0.4393 | 1.8299 |


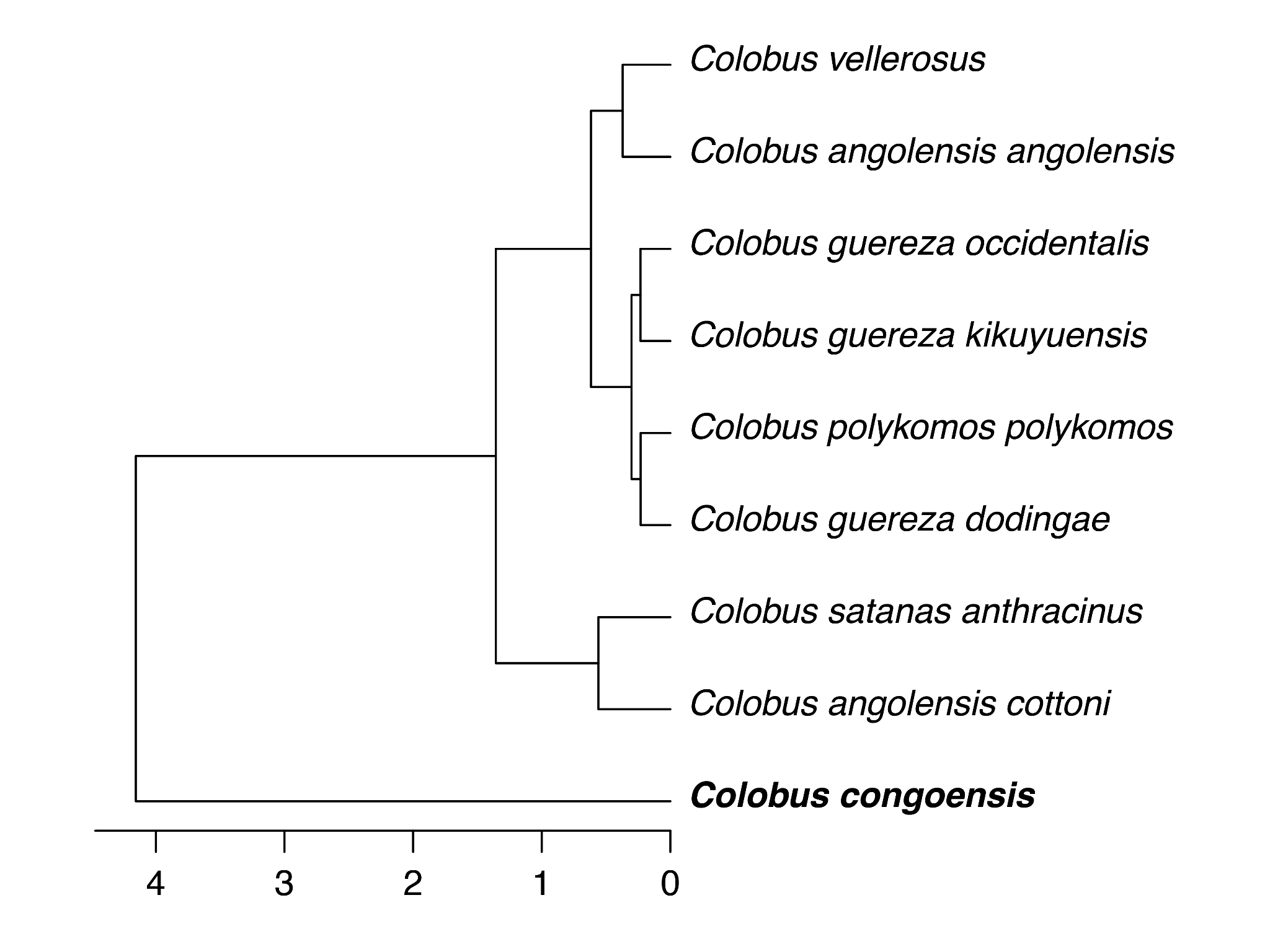


**Figure F**. UPGMA clustering dendrogram using the pairwise distance matrix of *Colobus* subspecies calculated from a pooled-sex sample of 8 craniomandibular indices (Table D). Dendrogram generated in R with *hclust()*. Note that *C. angolensis angolensis* is represented by a single male cranium, and *C. vellerosus* and *C. guereza dodingae* are each represented by a single female cranium. Scale reflects Euclidean distance. Cophenetic correlation coefficient = 0.9652.
