# Supplementary material for: Likweli: A remarkable new species of Colobus monkey from the Lomami National Park, Democratic Republic of Congo
Source: PLoS One. 2026 Jul 15;21(7):e0349857. doi: 10.1371/journal.pone.0349857 (PMC13372154; doi:10.1371/journal.pone.0349857)
Supplement: S5 File — (DOCX) [file pone.0349857.s005.docx]

S5 File: Supplementary phylogenetic trees and divergence date estimates


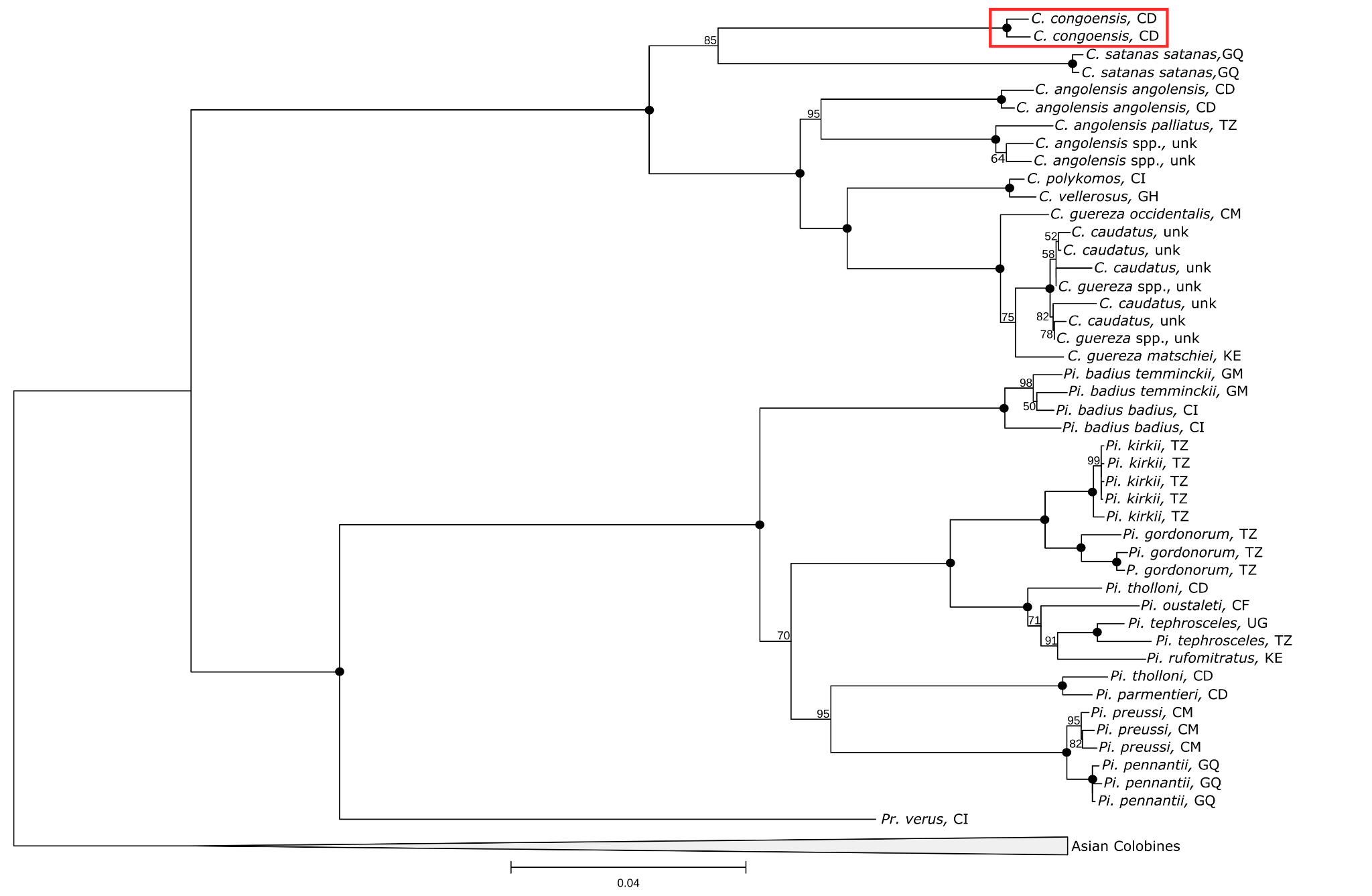


**Figure A. Maximum likelihood tree depicting the mitochondrial phylogenetic relationships among African colobines.** RaxML was used to infer phylogenetic relationships of African colobines. Numbers at nodes indicate bootstrap support values. Solid black circles denote nodes with 100% bootstrap support. The scale bar represents substitutions per site. The country codes are listed here: CD - The Democratic Republic of Congo, CF - Central African Republic, CI - Cote D’Ivoire, CM - Cameroon, GH - Ghana, GM - Gambia, GQ - Equatorial Guinea, KE - Kenya, TZ - Tanzania, UG - Uganda, unk - unknown.

| **Table A: Divergence dates (means) and 95% highest posterior density (HPD) intervals in millions of years (mya) using two different calibration dates.** | | | | |
| --- | --- | --- | --- | --- |
| Taxa groups | de Vries & Beck (2023) | | Perelman et al. (2011) | |
|  | Estimated divergence date | 95% HPD | Estimated divergence date | 95% HPD |
| *C. congoensis + C. satanas - C. angolensis + C. polykomos + C. vellerosus + C. guereza + C. caudatus* | 6.34 | 5.50-7.17 | 5.22 | 4.58-5.85 |
| *C. congoensis - C. satanas* | 5.02 | 4.27- 5.78 | 4.13 | 3.55-4.73 |
| *C. angolensis - C. polykomos + C. vellerosus + C. guereza + C. caudatus* | 3.99 | 3.42-4.58 | 3.28 | 2.84-3.76 |
| *C. angolensis palliatus - C. angolensis angolensis* | 3.44 | 2.90-4.00 | 2.83 | 2.41-3.29 |
| *C. polykomos + C. vellerosus - C. guereza + C. caudatus* | 3.15 | 2.67-3.68 | 2.60 | 2.20-3.02 |
| *C. polykomos - C. vellerosus* | 0.36 | 0.24-0.50 | 0.30 | 0.20-0.41 |
| *C. guereza - C. caudatus* | 0.84 | 0.64-1.07 | 0.69 | 0.53-0.88 |


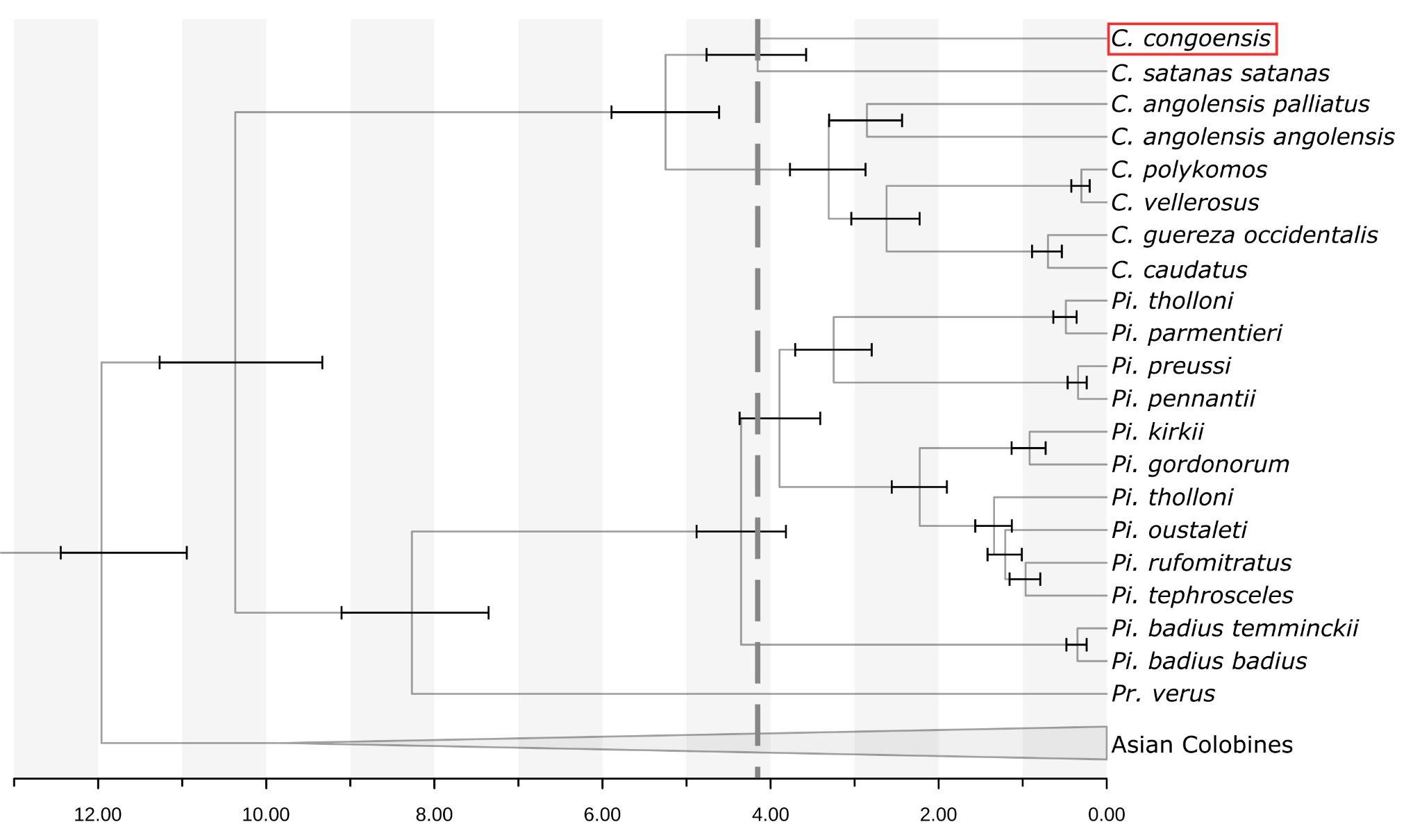


**Figure B.** **Estimated divergence dates of African colobines using a secondary calibration node.** MCMCTree divergence date estimates using available mitochondrial data and a secondary calibrated node based on estimated divergence dates from Perelman et al, 2011. The error bars on the nodes represent 95% highest posterior density intervals. The dashed gray line highlights the inferred divergence date between *C. congoensis* and *C. satanas*. *C. congoensis* is highlighted by a red rectangle.
