# Supplementary material for: Likweli: A remarkable new species of Colobus monkey from the Lomami National Park, Democratic Republic of Congo
Source: PLoS One. 2026 Jul 15;21(7):e0349857. doi: 10.1371/journal.pone.0349857 (PMC13372154; doi:10.1371/journal.pone.0349857)
Supplement: S1 Text — (PDF) [file pone.0349857.s007.pdf]

Inclusivity in global research

PLOS' policy on inclusivity in global research aims to improve transparency in the reporting of research performed outside of researchers' own country or community and ensures that PLOS publications reporting global research adhere to high standards for research ethics and authorship. Authors of relevant research articles may be asked to complete the questionnaire below, which outlines ethical, cultural, and scientific considerations specific to inclusivity in global research. This questionnaire may be requested when researchers have travelled to a different country to conduct research, if research uses samples collected in another country, research with Indigenous populations or their lands, or if research is on cultural artefacts. Researchers travelling to another country solely to use laboratory equipment will not normally be required to complete the questionnaire. However, the questionnaire can be requested at the journal's discretion for any submission – if you have been requested to complete this questionnaire by the PLOS journal you submitted to, please do so.

Please complete the questionnaire below and include this as a Supporting Information file with your manuscript. Note that if your paper is accepted for publication, this checklist will be published with your article in the supporting information files. Please ensure that you reference the checklist in the main body of your manuscript. We suggest adding a subsection 'Inclusivity in global research' to your Methods section and adding the following sentence: "Additional information regarding the ethical, cultural, and scientific considerations specific to inclusivity in global research is included in the Supporting Information (SX Checklist)"

The questions have been designed to be applicable to a wide range of study types, and there are subsections for both human subjects research and non-human subjects research. If any of the questions are not relevant to your research please mark them as "N/A" as appropriate.

Ethical considerations, permits and authorship

This section is applicable to all research types.

Provide details as to who granted permissions and/or consent for the study to take place in the Methods section of your manuscript. This should include the names of **all** ethics boards, governmental organizations, community leaders or other bodies that provided approval for the study. If individuals provided approval refer to these people by their role or title but do not list their name(s).

We reported all permissions granted in our research in Materials and Methods Sections 1 (Ethics statement), 2 (Local knowledge), and 3 (Inclusivity and global research).

If there were any deviations from the study protocol after approval was obtained please provide details of these changes in the Methods section of your manuscript.

N/A

Did this study involve local collaborators that are residents of the country where the research was conducted or members of the community studied? If you do not have any authors from said communities, please provide an explanation for this below.

Everyone listed as an author should meet PLOS' criteria for authorship and all individuals who meet these criteria should be included in the author byline, rather than the acknowledgements. For further information please see the journal's Authorship Policy.

Yes, three authors on this manuscript are citizens of the Democratic Republic of Congo and represent scientific, governmental, and community levels of involvement in the research.

Human subjects research (e.g. health research, medical research, cross-cultural psychology)

Did you obtain written informed consent from a representative of the local community or region before the research took place? How did you establish who speaks for the community? Details of written informed consent obtained from study participants should be reported separately in the Methods section of your manuscript.

Local individuals suggested places to look for the animals in question. They provided their own experiences with primates in general and their locations. The people "interviewed" were not part of a study specific to this paper, but among the questions asked some were pertinent to this paper and the answers are incorporated into the paper.

How did members of the local community provide input on the aims of the research investigation, its methodology, and its anticipated outcome(s)?

The persons whose observations are in this paper were not speaking for the community but rather as individuals from the area who had familiarity with the fauna and flora. The paper does not attempt to define community opinion. These “interviews” were part of an awareness raising mission in the northeast region of the buffer zone of Lomami National Park before the actual marking of park limits. A very large team, including many of the people “interviewed”, walked the border and participated in the marking. The prior awareness raising and interviews were to demonstrate that most of their land was in the buffer zone not the park, that the park’s border was fixed and would not move, that the buffer zone was rich in wildlife, and that they were free to hunt, fish, and farm in the buffer zone. ICCN (Parks Institute) wanted to know what was in the buffer zone and in so learning, give value to the villagers’ knowledge. The questions about the colobus monkeys were part of this exercise. There was no attempt or need to get “consent” from villages.

When engaging with the local community, how did you ensure that the informed consent documents and other materials could be understood by local stakeholders?

Although written consent was not required, we used Swahili and the local language (Mituku) to ask questions about the location and description of flora and fauna.

Will the findings of the research be made available in an understandable format to stakeholders in the community where the study was conducted (e.g. via a presentation, summary report, copies of publications, etc.)? Please provide details of how this will be achieved.

The finding of this research will be made available through the Lomami National Park outreach network and at academic institutions in DRC. More specifically, we will make a 15-minute film in the local language for the park management outreach team to use with local communities. Presentations will be given to Congolese universities, specifically the University of Kindu and University of Kisangani.

Non-human subjects research using specimens/ animals collected as part of the study, or those housed in archival collections. Examples include archaeology, paleontology, botany and zoology.

Did the permission you obtained from a local authority to perform the study include an agreement on access to outputs and benefit sharing? This may include procedures to enable fair distribution of the benefits and

resources arising from the research performed. Please include any details of Prior Informed Consent and Benefit Sharing Agreements obtained. These may be required by field-specific regulations, for example the Convention on Biological Diversity (CBD) and the associated Nagoya Protocol.

We obtained permissions to perform all aspects of the research from the Congolese Institute for Nature Conservation (ICCN), the DRC government agency responsible for protected areas and jurisdiction over their wildlife. The research in the study is the foundation for the PhD degree of one of the lead authors of the manuscript from the DRC.

If the material used in your study was imported, please A) provide the year it was imported and B) indicate whether permits were obtained to import/export the materials used, C) provide details of any permits obtained. If this information is not available, please indicate this.

Samples were imported in September 2021. We obtained permits from DRC CITES Authority, US Fish and Wildlife Service, and CDC to import skeletal, pelage, and tissue samples of colobus monkeys to the Yale Peabody Museum. CITES Permit Certificate No. CDFF0871R, CDFF0873R, CDFF0874R; PHS Permit No. 20210512-1878A. USFWS edec 2021954941.

If you used archival specimens, please state how the material used in your study was acquired by the institute it is held in and provide details of any permits obtained for the original excavations/ sample collection. If this information is not available, please indicate this.

Museum specimens used for the comparative skeletal study are all from public repositories that have searchable collections either via their online databases or VertNet. We provide all museum specimen IDs in Table A in S1 File.

How was the potential cultural significance of the materials collected in your study to local communities considered in your research design? Were Indigenous peoples and/or local researchers and institutions involved with archaeological excavations / collection of specimens? If so, please provide a description of their involvement.

N/A

If your manuscript includes photographs of human remains please indicate whether authors obtained permission from descendants or affiliated cultural communities to do so.

N/A
