# Supplementary material for: Likweli: A remarkable new species of Colobus monkey from the Lomami National Park, Democratic Republic of Congo
Source: PLoS One. 2026 Jul 15;21(7):e0349857. doi: 10.1371/journal.pone.0349857 (PMC13372154; doi:10.1371/journal.pone.0349857)
Supplement: S2 Table — (DOCX) [file pone.0349857.s009.docx]

**S2 Table:** Comparison of selected external morphological characters in adult *Colobus*.^1^

| **Species** | ***angolensis*** | ***guereza***^2^ | ***polykomos*** | ***vellerosus*** | ***satanas*** | ***congoensis* sp. nov.**^3^ |
| --- | --- | --- | --- | --- | --- | --- |
| Facial skin | Dark gray to black, glabrous; few fringing hairs. | Dark gray to black; covered with fine whitish gray hairs. | Black, glabrous; cheeks and chin with gray fringing hairs, nose dark gray. | Black, sparsely covered with fine white hairs. | Black, glabrous; orbits and zygomatic area gray, nose dark gray in some individuals. | Black, glabrous; cheeks and temporal region gray; skin under nose and around mouth creamy orange. |
| Pinnae | Black, glabrous; margin slightly irregular. | Black; margin not indented. | Black, glabrous; margin slightly irregular. | Black, glabrous; margin slightly irregular. | Large, black, glabrous; margin highly irregular. | Large, black, glabrous; margin irregular. |
| Tail | Variable by subspecies. Multicolored black, white, gray. Terminal tuft variably present. | Variable by subspecies. Multicolored black, white, gray. Terminal tuft present. | Largely white. Terminal tuft present. | Largely white, intermixed black hairs. Small terminal tuft present. | Black, scattered gray hairs on some individuals. Base of tail highly enlarged with basal tuft of long hairs. No terminal tuft. | Black, scattered gray hairs on some individuals. Base of tail minimally enlarged without basal tuft. Small terminal tuft in male. |
| Perineum and underside tail | White hair patch anterior to callosities in both sexes; extends ventrally in pubic band in males of some subspecies. | White hair patch anterior to and ringing callosities; separated in females, continuous in males. | White hair patch ventral to callosities; separated in female in two crescent zones, continuous in male. | White hair patch surrounds callosities; broken at perineum and underside of tail in female, continuous and extending ventrally in male. | No white hair patch; black perineal patch; swells in sexually active females. | White perianal skin patch; glabrous in female, covered with fine white hairs in male. |
| Penis | Pink | Pink | Pale gray to pink | Pink | Not described | Pinkish cream |
| Scrotum | Dark gray | Black, dark gray | Dark gray | Black | Dark gray | Dark gray |
| Callosities | Creamy orange to orange | Gray | Gray | Gray | Grayish orange | Creamy orange |

^1^ Sources for previously recognized species: Mittermeier et al. 2013. Cercopithecidae in Handbook of the Mammals of the World. Vol 3, Primates. Lynx Edicions, Barcelona. Pp 550-755; Rowe and Myers. All the World's Primates. <https://www.alltheworldsprimates.org/>; Butynski et al. 2013. Mammals of Africa, Vol. 2, Primates; Groves 2007; Journal of Anthropological Sciences Vol. 85 (2007), pp. 7-34; Butynski and de Jong. 2018. Geographic range, taxonomy, and conservation of the Mount Kilimanjaro guereza colobus monkey (Primates: Cercopithecidae: *Colobus*). *Hystrix* 29: 81–85; www.inaturalist.org

^2^ Includes *C. caudatus*.

^3^ Source of character traits for *C. congoensis* sp. nov.: this study.
