# Supplementary material for: Likweli: A remarkable new species of Colobus monkey from the Lomami National Park, Democratic Republic of Congo
Source: PLoS One. 2026 Jul 15;21(7):e0349857. doi: 10.1371/journal.pone.0349857 (PMC13372154; doi:10.1371/journal.pone.0349857)
Supplement: S5 Table — (DOCX) [file pone.0349857.s012.docx]

**S5 Table**: Distribution and endemicity of 15 species of primates in Lomami National Park located in the Tshuapa-Lomami-Lualaba (TL2) interfluves (see Text Figure 1).

| **Species** | **Interfluve zone** | | **Endemicity to DR Congo** | **Note** |
| --- | --- | --- | --- | --- |
|  | **WEST Lomami-**  **Tshuapa** | **EAST Lomami - Lualaba** |  |  |
| **Lorisidae** | | | | |
| *Perodicticus edwardsi* Central Potto | Present | Present | Non-endemic | Taxonomic identity in EAST interfluve is uncertain. |
| **Galagidae** | | | | |
| *Galagoides thomasi* Thomas’s Dwarf Galago | Present | Present | Non-endemic |  |
| *Galagoides demidoff* Demidoff’s Dwarf Galago | Present | Present | Non-endemic |  |
| **Hominidae** | | | | |
| *Pan paniscus* Bonobo | Present | Present | Endemic | Marked genetic differentiation between WEST and EAST interfluves. |
| **Cercopithecidae** | | | | |
| **Colobinae** | | | | |
| *Colobus congoensis* Likweli | Absent | Present | Endemic | Endemic to EAST interfluve. |
| *Colobus angolensis* Angolan Black and White Colobus | Present | Present | Non-endemic |  |
| *Piliocolobus tholloni*  Tshuapa Red Colobus | Present | Absent | Endemic |  |
| *Piliocolobus parmentieri*  Lomami Red Colobus | Absent | Present | Endemic |  |
| **Cercopithecinae** | | | | |
| *Lophocebus aterrimus* Black Mangabey | Present | Present | Near endemic |  |
| *Chlorocebus dryas* Dryad Monkey (Inoko) | Present | Present | Endemic |  |
| *Cercopithecus wolfi* Wolf’s Monkey | Present | Present | Endemic | *C. wolfi wolfi* WEST interfluve;  C*. wolfi elegans* EAST interfluve. |
| *Cercopithecus mitis* Blue Monkey | Present | Present | Non-endemic | Endemic subspecies, *Cercopithecus mitis heymansi.* |
| *Cercopithecus ascanius* Red tailed Monkey | Present | Present | Non-endemic |  |
| *Cercopithecus lomamiensis* Lesula | Present | Absent | Endemic | Endemic to WEST interfluve. |
| *Cercopithecus neglectus* De Brazza’s Monkey | Present | Present | Non-endemic |  |
| **Total Species** | **13** | **13** |  | **Total TL2 species: 15** |
